# Mixture model normalization for non-targeted gas chromatography/mass spectrometry metabolomics data

**DOI:** 10.1186/s12859-017-1501-7

**Published:** 2017-02-02

**Authors:** Anna C. Reisetter, Michael J. Muehlbauer, James R. Bain, Michael Nodzenski, Robert D. Stevens, Olga Ilkayeva, Boyd E. Metzger, Christopher B. Newgard, William L. Lowe, Denise M. Scholtens

**Affiliations:** 1 Department of Preventive Medicine, Division of Biostatistics, Northwestern University Feinberg School of Medicine, Chicago, IL 60611 USA; 2 Sarah W. Stedman Nutrition and Metabolism Center, Duke University Medical Center, Durham, NC 27701 USA; 3 Duke University School of Medicine, Durham, NC 27701 USA; 4 Department of Medicine, Division of Endocrinology, Northwestern University Feinberg School of Medicine, Chicago, IL 60611 USA

**Keywords:** Metabolomics, Non-targeted, Gas chromatography/mass spectrometry, GC/MS, Normalization, Batch effects

## Abstract

**Background:**

Metabolomics offers a unique integrative perspective for health research, reflecting genetic and environmental contributions to disease-related phenotypes. Identifying robust associations in population-based or large-scale clinical studies demands large numbers of subjects and therefore sample batching for gas-chromatography/mass spectrometry (GC/MS) non-targeted assays. When run over weeks or months, technical noise due to batch and run-order threatens data interpretability. Application of existing normalization methods to metabolomics is challenged by unsatisfied modeling assumptions and, notably, failure to address batch-specific truncation of low abundance compounds.

**Results:**

To curtail technical noise and make GC/MS metabolomics data amenable to analyses describing biologically relevant variability, we propose mixture model normalization (mixnorm) that accommodates truncated data and estimates per-metabolite batch and run-order effects using quality control samples. Mixnorm outperforms other approaches across many metrics, including improved correlation of non-targeted and targeted measurements and superior performance when metabolite detectability varies according to batch. For some metrics, particularly when truncation is less frequent for a metabolite, mean centering and median scaling demonstrate comparable performance to mixnorm.

**Conclusions:**

When quality control samples are systematically included in batches, mixnorm is uniquely suited to normalizing non-targeted GC/MS metabolomics data due to explicit accommodation of batch effects, run order and varying thresholds of detectability. Especially in large-scale studies, normalization is crucial for drawing accurate conclusions from non-targeted GC/MS metabolomics data.

**Electronic supplementary material:**

The online version of this article (doi:10.1186/s12859-017-1501-7) contains supplementary material, which is available to authorized users.

## Background

Non-targeted metabolomics technologies are unique tools in high-throughput ‘omics’ that provide an integrative measure of genetic and environmental factors contributing to metabolism and related phenotypes [1]. Techniques such as gas-chromatography/mass-spectrometry (GC/MS), liquid-chromatography/mass-spectrometry and nuclear magnetic resonance have their own strengths for varying applications, but all work toward the same goal to comprehensively characterize metabolite levels in samples of interest. These approaches are frequently accompanied by targeted technologies for which levels of specific metabolites are assayed and calibrated, for example by using stable isotope-labeled internal standards with an external series of unlabeled calibrants. When used for large-scale studies, non-targeted platforms generally require batching of samples over many days. Meaningful data analyses from large-scale studies demand careful application of quality control protocols for sample collection and storage, compound derivatization, metabolite extraction and reproducible annotation for all sample batches [2–5]. Even with precise monitoring of quality control procedures, large variations in metabolite abundance attributable to batch and run order within batch are well documented, particularly for GC/MS [1, 6]. In this manuscript, we propose a statistical approach to metabolomics data normalization to control technical variability attributable to batch and run order for large-scale metabolomics experiments. Data normalization is just one component of carefully crafted data quality pipelines that should be rigorously applied to minimize technical variability in large-scale metabolomics studies.

Many approaches for statistical control of batch- and run order-related technical variability, i.e. normalization, have been described [7–9]. Straightforward approaches calculate scaling factors, often based on total sample intensity or a relevant physiological variable, to be applied uniformly to all metabolites measured in a sample [10, 11]. While easy to use, these approaches do not account for the chemical diversity of all compounds and differential batch and run order effects often evident for different metabolites [1, 12]. Normalization approaches borrowed from gene expression microarray studies, including loess-based normalization [13], quantile normalization [14], surrogate variable analysis [15], empirical Bayes batch effect correction (ComBat) [16] and variance stabilizing normalization (VSN) [17] generally assume that few metabolites change across samples, that roughly equal numbers of metabolites are increased and decreased across samples, and/or that batch affects metabolites in similar ways. Any of these assumptions can be easily violated for metabolomics data [10]. Other approaches rely on addition of single [7] or multiple internal standard compounds [6] or a priori identification of a set of metabolites expected not to change in the experimental conditions [18]. Selection of these standards and non-changing sets could vary substantially depending on sample type and metabolite classes of interest. Furthermore, in sample types that are poorly understood, selected internal standards or non-changing compounds may not correspond well in terms of retention time and mass spectrometry peak alignment with metabolites observed in samples of analytical interest [12].

Noting chemical diversity of batch and run-order effects and the difficulty of a priori selection of internal standards, repeated assay of quality control (QC) samples from a consistent control pool is increasingly applied in large-scale metabolomics studies [1, 12, 19, 20]. While QC-based normalization methods are gaining favor, current approaches do not formally model well-known variation in thresholds of detectability across batches for GC/MS data [21, 22] and instead require elimination or imputation of abundance levels for undetected metabolites. This is particularly problematic for methods that rely on total compound abundance since low abundance compounds can be systematically missed in batches with higher detectability thresholds.

We describe a mixture model approach for non-targeted GC/MS metabolomics data normalization (mixnorm) that is compound-specific, avoids a priori selection of internal standard compounds, and formally models not only batch and run order effects, but also varying thresholds of detectability across batches. The estimated parameters for batch and run order effects account for truncation of undetected abundance levels in QC samples and are easily interpretable given their regression-based derivation. Mixture modeling has been used for downstream data analyses to investigate biological associations between phenotypes and metabolites [23]; in this application, we discuss an alternative use of mixture modeling for normalization purposes. A large-scale simulation study confirms accuracy of mixnorm over other methods for controlling technical variability and for detecting true associations with a simulated phenotype variable over a range of batch-specific detectability thresholds and undetected metabolites. Improved performance of mixnorm is also demonstrated using GC/MS data from 162 metabolites with reliable annotation in a reproducible AMDIS-based pipeline for 300 QC and 1200 analytical serum samples processed with highly standardized quality control procedures in the ongoing Hyperglycemia and Adverse Pregnancy Outcome (HAPO) Metabolomics study [24]. When evaluated according to variability of individual metabolites across QC and analytical samples, pairwise Spearman correlation of QC samples, and Spearman correlation with targeted assays of the same compounds, mixnorm demonstrates superior performance to other approaches evaluated here.

## Methods

### Mixture model normalization (mixnorm)

Mixture model normalization (mixnorm) uses data from QC samples drawn from a common pool and included at multiple run order positions within all GC/MS batches. Given their common source, observed systematic variation in abundance levels for a given metabolite for QC samples is attributable to batch and/or run order within batch. If multiple QC types are used, it can also be assumed that batch- and run-order effects are equal across QC types for detectable metabolites, even if the actual abundance levels vary. Mixnorm uses QC data to estimate batch- and run order effects, and then applies these corrections to samples of analytical interest to prepare data for downstream analysis.

The mixture model adopted by mixnorm jointly models batch and run order as they pertain to metabolite detectability in QCs and, if detected, abundance level. Importantly, data truncation for low abundance compounds is modeled using batch-specific thresholds. Adopting a model formulated in the context of antibody response to vaccine [25], the following specifies the mixture model likelihood contribution for the *i*th QC sample for a given metabolite under analysis:

$$ {\left(\left(1-{p}_i\right)+{p}_i\varPhi \left[\left({T}_i-{\mu}_i\right)/\sigma \right]\right)}^{\left(1-{\delta}_i\right)}{\left({p}_i\cdot \exp \left[-{\left({y}_i-{\mu}_i\right)}^2/2{\sigma}^2\right]/\sqrt{2\pi \sigma}\right)}^{\delta_i} $$

where *p*_*i*_ represents the probability of metabolite presence in the *i*th sample, T_*i*_ is the threshold of detectability for the *i*th sample, *μ*_*i*_ is the true mean level of the metabolite in the *i*th sample, σ^2^ is the variance of the metabolite, δ_i_ is an indicator equal to 1 if the metabolite is detected and 0 otherwise, *y*_*i*_ is the observed level of the metabolite if it is detected and ϕ is the normal cumulative distribution function (cdf).

The first component of the likelihood,

$$ {\left(\left(1-{p}_i\right)+{p}_i\varPhi \left[\left({T}_i-{\mu}_i\right)/\sigma \right]\right)}^{\left(1-{\delta}_i\right)}, $$

contributes when δ_i_ = 0, i.e. when a metabolite is not detected in the *i*th QC sample. A metabolite may not be detected either because it is truly absent from the sample or because it is present below the detectability threshold. Mixnorm specifies a logistic model for *p*_*i*_ as log(*p*_*i*_ */*(1-*p*_*i*_)) = *x*_*i*_ *’β*, where *x*_*i*_ and *β* are covariate and parameter vectors, respectively. Including (1-*p*_*i*_) in this component of the likelihood allows for the small probability that a metabolite would degrade over the course of running a batch and would therefore be undetectable due to true absence from the sample. The remainder of the first component of the likelihood models the probability that the metabolite is present in the sample but below the detectability threshold T_*i*_ using a normal cdf ϕ. T_*i*_ is specified in mixnorm as the minimum observed metabolite abundance for the batch that included sample *i*. Mixnorm specifies a linear model for the mean of the metabolite level, *μ*_*i*_ with *μ*_*i*_ = *z*_*i*_ *’α*, where *z*_*i*_ and *α* are covariate and parameter vectors, respectively. Mixnorm assumes that, conditional on technical covariates relevant for normalization, the variance σ^2^ of metabolite levels in QCs is the same across batches.

The second component of the likelihood,

$$ {\left({p}_i\cdot \exp \left[-{\left({y}_i-{\mu}_i\right)}^2/2{\sigma}^2\right]/\sqrt{2\pi \sigma}\right)}^{\delta_i}, $$

contributes when δ_i_ = 1, i.e. when a metabolite is detected in the *i*th QC sample and abundance is quantified as log2-transformed MS peak area. This component of the likelihood models the probability that the metabolite is present *p*_*i*_ and specifies a normal distribution with mean *μ*_*i*_ and variance σ^2^ for the observed value *y*_*i*._ The logistic and linear regression models described above for *p*_*i*_ and *y*_*i*_ link the two components of the likelihood.

Importantly, for normalization purposes, the covariates used to model variation in QC data should reflect technical factors, for example batch, run order within batch, or different types of QC pools. While covariate vectors *x*_*i*_ and *z*_*i*_ can be specified to include the same covariates, mixnorm does not require that they be identical. A more limited set of variables could be appropriate for *x*_*i*_ depending on the number of QC samples and the frequency of undetected metabolites.

Maximum likelihood parameters are estimated in mixnorm using BFGS optimization over all QCs. After estimating model parameters *β* and *α*, location shift corrections are applied to observed metabolite levels for all QCs and to samples of analytical interest, according to effect estimates and covariates for each sample. In experiments that include multiple QC types, mixnorm will estimate the mean difference in metabolite levels for different types of QCs. If different QC pools are reflective of different types of analytical samples of interest, these location shifts can be applied to analytical data if desired. Mixnorm functionality is available in the metabomxtr R package (devel) [23] at http://www.bioconductor.org/ [26].

### Other normalization methods

Normalization methods compared to mixnorm in this study are described briefly below, with more lengthy descriptions and a table comparing features in Additional file 1.

#### Mean centering

For each metabolite, the difference between the batch-specific mean and the mean across all samples in all batches for that metabolite is subtracted from the observed metabolite level.

#### Median scaling

For each metabolite, the abundance level in a given batch is divided by the ratio of its batch-specific median to the median for that metabolite across all samples in all batches.

#### Quantile normalization

Quantile normalization uses the means of ranked values within samples to match the distribution of abundance levels across all samples [14].

#### Quantile + ComBat

Quantile normalization is followed by ComBat, an empirical Bayes method using metabolite-specific estimates of mean and variance to correct for batch, while maintaining phenotype effects [16]. ComBat requires complete data, therefore missing values are imputed using Bayesian principle components (PC) analysis with half-minimum value substitutions for negative imputed values [27].

#### EigenMS

EigenMS first requires estimation of a categorical ‘treatment’ effect via ANOVA. Singular value decomposition is then applied to the matrix of residuals and additional bias trends are removed from the data [28].

#### Batch Normalizer

Batch Normalizer is a regression-based algorithm that relies on QC samples and incorporates total abundance of each sample when estimating corrections for batch and run order effects [20].

#### Variance Stabilizing Normalization (VSN)

VSN applies a smooth transformation to all metabolites that mimics a log transformation for high intensity values and linear scaling for low intensity values, rendering variance approximately constant across the full range of intensities [17].

#### Software

Mean centering, median scaling and Batch Normalizer were implemented using R; functions are available in Additional file 2. Quantile normalization was implemented using preprocessCore (version 1.36.0) [29] R package. Quantile + ComBat used preprocessCore (version 1.36.0) [29] and sva (version 3.22.0) [30] R packages, with Bayesian PC imputation from pcaMethods (version 1.66.0) [27] R package. VSN was implemented using vsn (version 3.42.3) [17] R package. PreprocessCore, sva, pcaMethods and vsn are all available at http://www.bioconductor.org/ [26]. EigenMS R functions are available at http://www.sourceforge.net/ [28].

### Simulation study

We conducted a simulation study to assess mixnorm’s performance relative to other normalization approaches. We simulated a GC/MS experiment with 150 metabolites for 20 batches of 24 analytical and 3 QC samples each, totaling 480 analytical and 60 QC samples. Each metabolite was assigned a mean ‘intercept’ *α*_*m*_ (*m* = 1,…,150) according to a random draw from a normal distribution with mean 18 and standard deviation 2, *α*_*m*_ *~* N(18,2^2^), placing the simulated metabolite means within the range of 13.5–23.5, consistent with typical GC/MS log2 transformed peak areas. Each analytical sample was next assigned a ‘phenotype’ *v*_*i*_ (*i* = 1,…,480) according to a random draw from a standard normal distribution, *v*_*i*_ ~ N(0,1). Phenotype associations varied according to *β*_*m*_ (*m* = 1,…,150) for each metabolite, with *β*_*m*_ sampled from a standard normal distribution, *β*_*m*_ ~ N(0,1). Values of *α*_*m*,_ *v*_*i*_ and *β*_*m*_ were held constant for 1000 simulation rounds.

In each round, prior to including batch effects, the abundance level *z*_*jm*_ for QC sample *j* for metabolite *m* was specified *z*_*jm*_ *= α*_*m*_ + *ε*_*jm*_, where *ε*_*jm*_ ~ N(0,(.03*α_*m*_)^2^). The abundance level *y*_*im*_ for analytical sample *i* for metabolite *m* included the association with phenotype and was specified *y*_*im*_ *= α*_*m*_ *+ β*_*m*_ *v*_*i*_ + *ε*_*im*_ *,* with *ε*_*im*_ ~ N(0,(.03*α_*m*_)^2^). Consistent with QC samples generated by pooling equal volume aliquots from all analytical samples, our QC sample means for all metabolites are those expected at the ‘mean’ phenotype of 0 for analytical samples. Prior to introducing batch variability, these simulation parameters yield relative standard deviations (RSDs, standard deviation for a metabolite divided by its mean) of roughly 3% (ranging 1.77 to 4.32%, Table 1) for all metabolites in QC samples and higher RSDs averaging 5.82% (ranging 2.65 to 16.15%, Table 1) for analytical samples. As expected in an experimental setting, analytical samples include variability attributable to associations with phenotype thus analytical sample RSDs are higher.

**Table 1:** Summary statistics for metabolite variability according to RSD for QC and analytical samples prior to and following normalization

	RSD % of individual metabolites across samples: mean (min, max)	
	QC	Analytical
Truth	2.99 (1.77, 4.32)	5.82 (2.65, 16.15)
Not normalized	10.03 (2.33, 18.81)	10.88 (2.88, 19.04)
Mean centering	3.08 (1.09, 6.08)	5.24 (1.81, 14.72)
Median scaling	3.08 (.97, 6.30)	5.32 (1.84, 14.97)
Quantile	10.21 (1.02, 18.69)	10.66 (2.29, 18.27)
Quantile + ComBat	3.94 (1.10, 11.70)	5.65 (1.62, 19.01)
EigenMS	6.82 (1.75, 15.27)	7.05 (1.76, 16.05)
VSN	9.99 (2.27, 17.51)	10.84 (2.81, 19.27)
Batch normalizer	1.73 (.19, 3.09)	6.26 (2.41, 16.71)
mixnorm	2.42 (.73, 4.59)	5.81 (1.84, 19.62)

In GC/MS, batch effects vary depending on chemical class and don’t necessarily follow monotonic trends over all batches. In each simulation round, we therefore randomly sampled batch effects for metabolite *m* in batch *k*, *b*_*mk*_ ~ N(0,2^2^). These batch effects were then added to QC and analytical sample levels such that if QC sample *j* was in batch *k*, *z*_*jm*_ *= z*_*jm*_ *+ b*_*mk*_ and if analytical sample *i* was in batch *k*, *y*_*im*_ *= y*_*im*_ *+ b*_*mk*_ for metabolite *m*. After generating simulated abundance levels, to mirror detection threshold variability across batches, we applied detection thresholds ranging incrementally from 12.5 to 15 and randomly applied across batches 1 to 20. Simulated values for all metabolites that fell below batch-specific detection thresholds were treated as undetected.

This simulation approach included batch effects with equal means for a given metabolite for QC and analytical samples assigned to the same batch, and the same detection threshold for all metabolites for a given batch. Once batch effects and batch-specific detection thresholds were included, RSD summarized over all QC and analytical samples increased as expected (Table 1). The increased variability caused by batch effects is precisely the technical noise that normalization seeks to control; i.e. RSD for correctly normalized data should equal RSD for simulated data prior to including batch effects. All simulated data, including unnormalized data and data after normalization using mixnorm and the other approaches described here, are publicly available at https://dataverse.harvard.edu/dataverse/gcmsmetab.

### Simulation data normalization metrics

All described normalization algorithms were applied to the simulated data. For mixnorm, covariates for batch were included in both the logistic and linear components of the model. Normalization results for simulated data were evaluated using RSD and associations with the simulated phenotype.

#### Relative Standard Deviation (RSD)

RSD was calculated for each metabolite prior to and after normalization. To assess consistency of RSDs after normalization with true RSDs prior to introducing batch effects and truncation in the simulation, we used simple linear regression with intercept term set to 0 for all metabolites in simulated analytical samples, treating estimated RSD as the outcome and true RSD as the predictor. Beta = 1 from this simple no-intercept linear regression model indicates perfect agreement of true and estimated RSD following normalization, with beta values lower (higher) than 1 indicating under- (over-) estimation of RSD after normalization. These linear regression analyses were examined for metabolites with varying proportions of undetected values. Metabolites were grouped by 0, 0–5%, 5–10%,…, 75–80% undetected values. Metabolites with >80% undetected values were omitted from analysis.

#### Detectable associations with simulated phenotype

Detectability of metabolite associations with the simulated phenotype variable *v*_*i*_ were summarized prior to and following normalization. The frequency of true positive and false positive associations were calculated for a range of values for *β*_*m*_ specified in the simulation.

### HAPO Metabolomics study

The original HAPO Study was an international population-based study conducted 2000–2006, designed to examine associations between maternal glucose levels during pregnancy and newborn outcomes. HAPO Study methods were described previously [31, 32]. The HAPO Study protocol was approved by the institutional review board at each HAPO field center and all participants provided informed consent. Over 23,000 eligible women at 15 international field centers underwent a 75-g oral glucose tolerance test (OGTT) between 24 and 32 weeks’ gestation. Fasting and 1-hour plasma glucose were measured and additional serum samples collected and stored using highly standardized protocols after rigorous training at all HAPO field centers [31, 32]. Immediately following collection, maternal and offspring cord serum samples were processed, stored at -20C or -80C for 1–6 weeks, shipped on dry ice to the HAPO Central Laboratory, and remained frozen at -80C until the present assays.

#### HAPO Metabolomics experimental design

HAPO Metabolomics was designed to study maternal and newborn metabolic profile associations with maternal glucose levels during pregnancy and newborn outcomes [24, 33]. Fasting and 1-hour maternal and newborn cord serum triples for 400 European ancestry mothers and their newborns were sampled for HAPO Metabolomics to reflect the distributions of characteristics observed in the original HAPO Study. Maternal serum samples at fasting and 1-hour following Trutol consumption during the OGTT and cord serum from their newborns were analyzed using conventional, targeted amino acid and non-targeted GC/MS metabolomics.

**Fig. 1:**
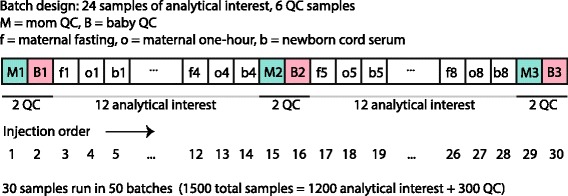
Schematic representation of run order within batch for the HAPO Metabolomics study. Data include 1200 analytical samples (400 maternal fasting, 400 maternal 1-hour, 400 newborn cord serum) of interest and 300 QCs (150 maternal, 150 newborn) processed in 50 batches of 30 samples each. Maternal samples placed at the beginning, middle and end of each batch are labeled M1, M2 and M3, respectively. Newborn (or baby) samples placed at the beginning, middle and end of each batch are labeled B1, B2 and B3, respectively. In a batch of total size 30, maternal QCs were placed at run order 1, 15 and 29 and newborn QCs were placed at run order 2, 16 and 30. Maternal / newborn sample triples were run in sequence with 8 sets of triples included in each batch

For GC/MS, HAPO Metabolomics samples were batched into sets of 24 comprised of fasting and 1-hour maternal and newborn cord serum from 8 mother-newborn pairs. Sample triples were randomly assigned to batches to balance phenotypic variables including maternal age, glucose, body mass index and newborn birth weight and sum of skinfolds. Two sets of QC sera were created by drawing 20-μL volumes from all HAPO Metabolomics analytical samples before analysis, combining these into separate pools for maternal and newborn sera, and splitting into 100-μL aliquots. After maintenance of GC and MS, QC samples were injected to passivate working surfaces of the instrument until chromatography and MS response were stable. After each re-tuning of the MS, adequate sensitivity was confirmed using a perfluorinated tributylamine tuning standard. Aliquots from each QC pool were run at the beginning, middle and end of each batch, yielding 30 samples total per batch (24 analytical samples and 6 QCs). Over a run of 30 samples within each batch, maternal QCs were run at positions 1, 15 and 29 and newborn QCs were run at positions 2, 16 and 30. A total of 50 batches were run totaling 1500 samples (1200 analytical samples and 300 QCs). Figure 1 illustrates the HAPO Metabolomics batching scheme.

#### Conventional metabolite and targeted amino acid assays

Conventional metabolites were measured on a Beckman-Coulter DxC600 autoanalyzer using reagents from Beckman (Brea, CA; lactate) and Wako USA (Richmond, VA; beta-hydroxybutyrate). For free glycerol, reagents by Roche (Indianapolis, IN) for glycerol-blanked triglycerides were modified. To 84 mL of the Roche R1 reagent, 6.0 mg 4-aminoantipyrine dye (Sigma, St. Louis, MO) was added. This assay was run by combining 250 μL reagent with 20 μL sample volume, calibrated against a glycerol standard (2.29 mM) with detection at 520 nm after 5 min. Targeted assays of amino acids using stable-isotope-labeled internal standards were performed on an Acquity TQD Triple Quadrupole system (Waters Corporation, Milford, MA). Absolute metabolite concentrations were calculated based on the linear relationship between concentration and peak area, and calibrated against internal standards with known concentrations, as previously described [34, 35]. Conventional metabolite and targeted amino acid data are used here to evaluate whether each normalization method can improve correlation of non-targeted GC/MS data with targeted measurements that are quantified using internal standards and not subject to batch effects.

#### Non-targeted GC/MS assay sample preparation and quality control

For non-targeted assays, serum was uniformly prepped for each batch using a modification of previously described protocols [36, 37]. Methanol, the extraction solvent, was spiked with a retention-time-lock (RTL) internal standard of perdeuterated myristic acid. Extracts were dried, and then prepared for non-targeted GC/MS by methoximation and trimethylsilylation [36], and run on a 6890N GC-5975 Inert MS (Agilent Technologies, Santa Clara, CA). Programmed-temperature vaporization in the inlet and post-run, mid-column, hot backflushing of the GC column minimized analyte decomposition, carryover, and fouling of GC and MS.

#### Non-targeted GC/MS peak deconvolution and annotation

GC/MS data were deconvoluted with AMDIS freeware, courtesy of National Institute of Standards and Technology, Gaithersburg, MD [38] and parsed against peaks annotated using the Fiehn RTL spectral library [36] with additions from our laboratory. Detected peak areas were log2-transformed for abundance quantification. Manual curation included re-annotating features that matched multiple metabolites from our library (often co-eluting isomers such as aldohexoses), and favoring those with higher AMDIS Reverse scores. Annotation was performed simultaneously for the full data set, so there were no inconsistencies across HAPO Metabolomics samples. Reliably annotated peaks for 162 unique metabolites with detected abundance levels in at least 20% of all samples were used in this analysis. All targeted and non-targeted HAPO Metabolomics data used in this manuscript are included in Additional file 3.

#### HAPO metabolomics GC/MS data normalization parameters

For HAPO Metabolomics GC/MS data normalization using mixnorm, in the logistic model for *p*_*i*_, we included indicator variables for batch (using the batch with median abundance level for the metabolite being normalized as the referent) and QC sample type (newborn v. referent maternal). For the linear regression component for *μ*_*i*_, we included batch and QC sample type, as well as log-transformed run order (the best fit to the data after exploring linear, quadratic and log-transformed effects). All other normalization methods were implemented as described in Additional file 1.

### HAPO metabolomics GC/MS data normalization metrics

#### Individual metabolite variability across QC samples

To view metabolite variability across QC samples, scatterplots were created with metabolite levels on the y-axis versus batch number on the x-axis with different plotting characters for maternal and newborn QCs and batch position. Undetected metabolites were indicated by a point below a dashed black line set below the minimum observed level for the metabolite of interest. Variability of detected metabolites was summarized as RSD across all maternal and newborn QC samples.

#### Individual metabolite variability across analytical samples

RSDs of metabolites across maternal fasting and 1-hour and newborn cord serum analytical samples were calculated prior to and following normalization. It is expected that RSD across all analytical samples of a given type would be higher than RSD across QC samples since analytical samples include biologically relevant variability.

#### Pairwise correlations of QC samples

To evaluate comparability of all metabolites in QC samples, pairwise Spearman correlations of maternal and newborn QC metabolites were calculated prior to and following normalization.

#### Correlations of non-targeted data with conventional and targeted amino acid data

Spearman correlation coefficients were calculated for non-targeted metabolites and their conventional metabolite and targeted amino acid counterparts on all HAPO Metabolomics analytical samples prior to and following normalization.

#### Associations with HAPO phenotypes

Associations with maternal fasting plasma glucose (FPG) in HAPO were modeled using metabolomics data from maternal fasting samples. Associations were modeled using two approaches. The first used linear regression, dropping unobserved metabolite levels from analysis or using imputed data as indicated by the normalization method. The second analysis approach used mixture modeling for downstream analysis. While similar in concept to the mixture model proposed here for normalization purposes, downstream mixture modeling is applied subsequent to normalization and the covariates used for downstream analysis include phenotypic predictors of interest. In both the linear regression and mixture model analyses, the primary covariate of interest was maternal FPG, but all analyses additionally included adjustment for HAPO Study field center (Belfast UK, Brisbane and Newcastle, Australia), maternal BMI, mean arterial pressure, maternal age and gestational age at OGTT, and sample storage time. The number of statistically significant associations with nominal *p* < 0.05 was summarized for analyses of HAPO Metabolomics GC/MS data after application of each normalization method. Pathway analyses using MetaboAnalyst 3.0 (http://www.metaboanalyst.ca/) were also conducted using hypergeometric tests to evaluate pathway enrichment of metabolites significantly associated with FPG [39].

## Results

### Simulation results

#### Relative Standard Deviation (RSD)

RSDs were calculated for simulated QC and analytical sample data prior to including batch effects (‘truth’), after including batch effects and truncation according to batch-specific thresholds (‘not normalized’) and after application of each normalization method (Table 1). While mixnorm slightly underestimates true RSD in QCs, the mean RSD for analytical samples of 5.81% is remarkably consistent with the true analytical sample mean RSD of 5.82%. Mean centering, median scaling and quantile + ComBat also yield RSDs that are similar to true RSDs, although the means are somewhat smaller and may indicate underestimation of true variability. Batch Normalizer RSDs for QC samples are quite low (mean 1.73%), although the mean for analytical samples is fairly consistent with the truth (mean 6.26%). Summary statistics for RSDs after quantile normalization, EigenMS and VSN suggested poorer correction of batch effects than the other methods.

**Fig. 2:**
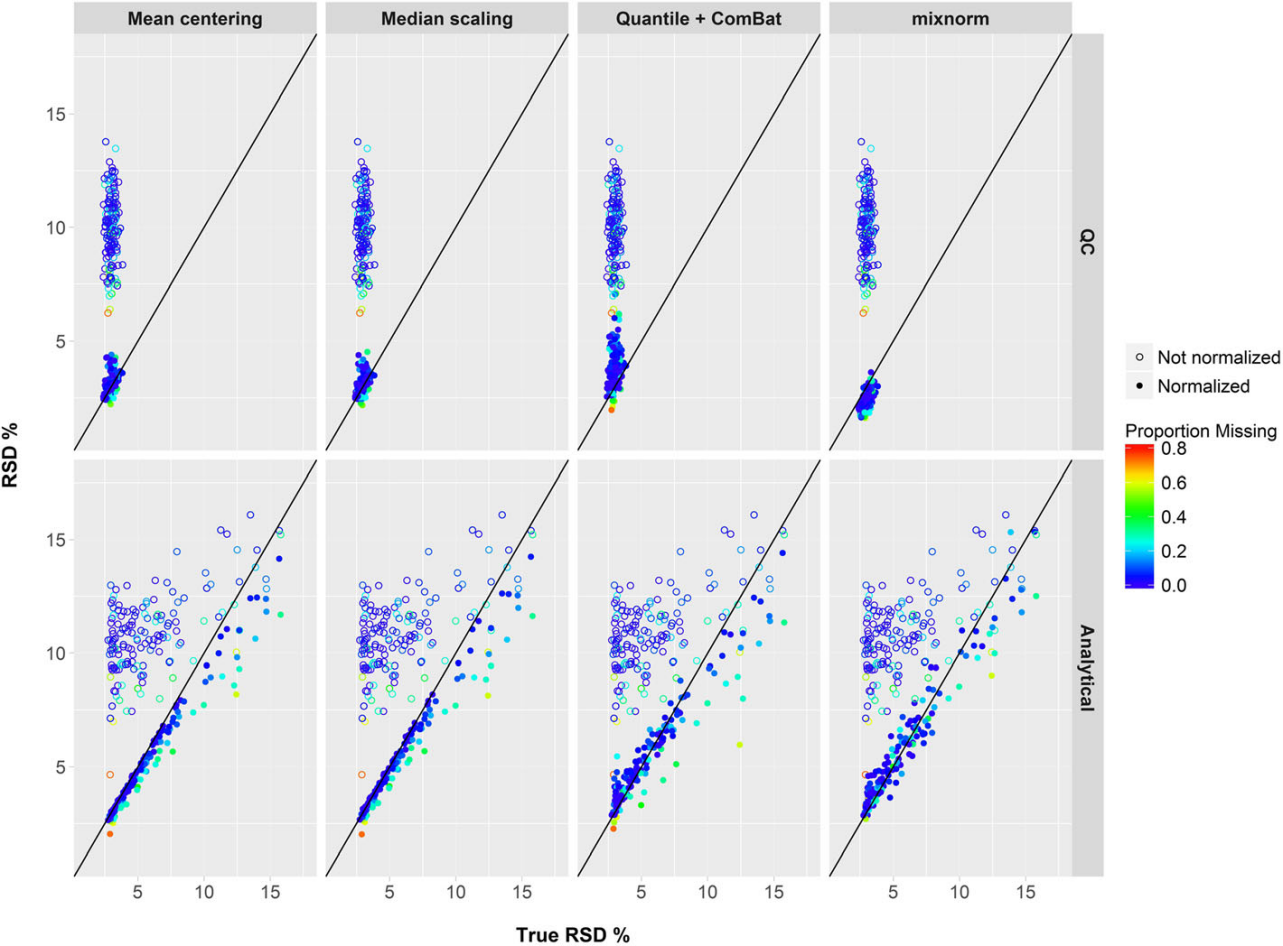
An example of one round of simulation results (simulation 316) comparing calculated RSD for metabolites in QC and analytical samples before normalization (*open circles*) and RSD after normalization for four different methods (*closed circles*) v. true RSD prior to inclusion of batch effects and batch-specific detection thresholds in the simulation. Points are colored according to the proportion of undetected levels in the simulation for that metabolite. The *black line* indicates perfect correspondence of true and estimated RSD

A unique feature of mixnorm compared to other methods is explicit modeling of data truncation due to batch-specific detection thresholds. Figure 2 plots RSD prior to and following normalization by mean centering, median scaling, quantile + ComBat and mixnorm v. true RSD for one set of simulated metabolite values (simulation round 316). Overall improvement in RSD after normalization for both QC and analytical samples is evident for all methods. As the proportion of undetected values increases, however, visual inspection of simulated analytical samples suggests that RSD is underestimated by mean centering, median scaling and quantile + ComBat more than mixnorm. Similar plots for nine other randomly selected simulation rounds results are included in Additional file 4: Figures S1–S9.

**Fig. 3:**
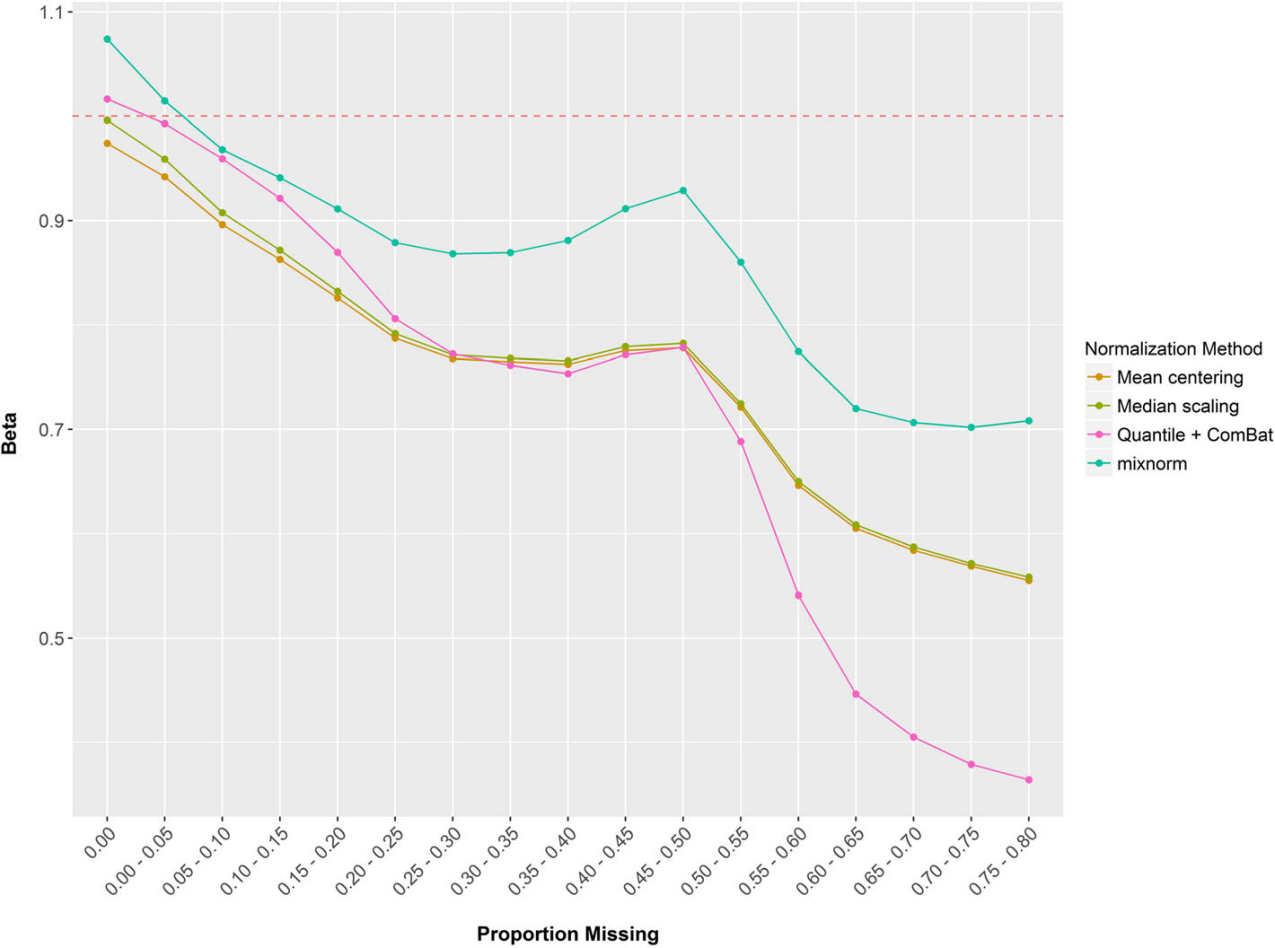
A plot of beta estimates from simple no-intercept linear regression models using simulation data. Calculated RSD after normalization was treated as the outcome and true RSD prior to inclusion of batch effects and batch-specific detection thresholds in the simulation was treated as the predictor. A beta value of 1 indicates perfect correspondence with beta values <1 (>1) indicating under- (over-) estimation of RSD by the normalization method. Betas are plotted according to increasing amounts of missing data, i.e. the proportion of simulated undetected values for a given metabolite

To summarize RSD estimates over all simulations for increasing amounts of undetected or ‘missing’ values, we examined beta estimates from simple no-intercept linear regression models treating estimated RSD after normalization as the outcome and true RSD as the predictor. Beta values for mixnorm, mean centering, median scaling and quantile + ComBat, the strongest methods in overall evaluations of RSD in the simulation, are plotted in Fig. 3. Beta values for all four methods are roughly equal to 1 for metabolites with no undetected values. As the proportion of undetected values increases, beta values fall below 1, with RSD underestimated by approximately 20% (beta = .8) for mean centering, median scaling and quantile + ComBat when 20-25% of metabolite levels are undetected. In contrast, beta values for mixnorm only decrease to .8 when more than 55% of metabolite levels are undetected. True RSD is more accurately recovered using mixnorm even when a metabolite is undetected in over half of the samples.

**Fig. 4:**
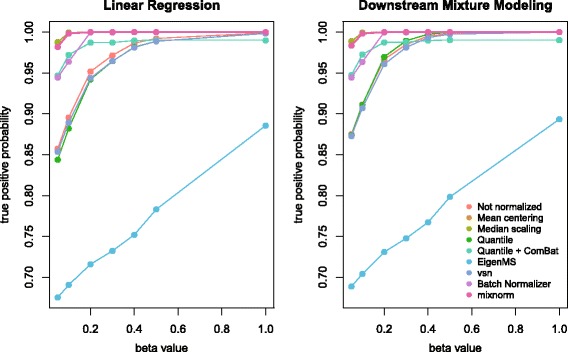
Plots of true positive probabilities (y-axis) under both linear regression and downstream mixture model analyses for detecting true associations in simulated data prior to and following normalization. Values on the x-axis represent the magnitude of association with the simulated phenotype according the simulated beta values. True positive probabilities are plotted for beta values with absolute value greater than or equal to 0.05, 0.1, 0.2, 0.3, 0.4, 0.5 and 1.0

Figure 4 illustrates results from association analyses with the simulated phenotype after normalization with each method (numeric results are reported in Additional file 5). Association analyses were conducted using linear regression either ignoring missing data or using imputed values depending on the normalization method, as well as downstream mixture modeling, i.e. a mixture model to accommodate both undetected and detected metabolite levels when identifying associations with phenotypes after QC-based normalization. For simulated beta values with absolute value greater than or equal to the values plotted on the x-axis, the probability of detecting these true associations with nominal *p* < 0.05 using both analytic approaches was plotted on the y-axis. Results are similar for linear regression and mixture modeling, with some increase in the true positive probability observed for mixture modeling due to explicit modeling of truncated data [23]. In general, mixnorm, mean centering and median scaling perform comparably in terms of most accurately identifying associations with at least .97 true positive probability for values of beta ≥0.05. Notably, the most distinct improvements in true positive probabilities occur for lower betas that correspond to more modest associations, underscoring the importance of controlling technical variability to detect modest effects that may otherwise be hidden by technical noise.

False positive probabilities were calculated by determining the frequency with which each method led to detection of associations for beta values approaching 0 (Additional file 5). These probabilities were comparable for mixnorm, mean centering and median scaling, with substantial increases in false positive probabilities observed for all other methods.

### HAPO Metabolomics results

#### Individual metabolite variability in QC samples

**Fig. 5:**
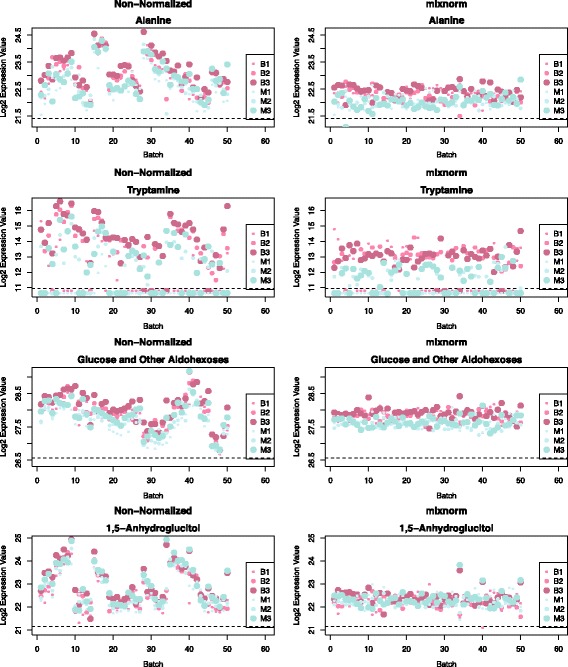
Log 2 peak areas for QC samples in HAPO Metabolomics across all 50 batches. Data are presented for peaks annotated as alanine, tryptamine, glucose and other aldohexoses and 1,5-anhydroglucitol. The first column contains original non-normalized observations and the second column contains mixnorm-normalized values. Small, medium and large blue dots correspond to maternal QC samples placed at the beginning (M1), middle (M2) and end (M3) of each batch, respectively. Small, medium and large pink dots correspond to newborn QC samples placed at the beginning (B1), middle (B2) and end (B3) of each batch, respectively. Dots below the dotted line represent values below the detection threshold for a given batch

Prior to normalization, metabolite levels across QC samples varied substantially by batch and run order (Fig. 5, first column). The plot of alanine demonstrates changes in observed abundance with each batch, with the largest jumps from one batch to the next (e.g. batch 27 to 28) often coinciding with routine cleanings. When detectable, tryptamine levels show trends similar to those for alanine, but since tryptamine is less abundant than alanine, for several batches tryptamine was not detected in QC samples. Variation in detectability thresholds across batches is evident for this metabolite given that some, but not all, batches exhibited undetectable values. Tryptamine was detectable in 64 (42.67%) and 111 (74.00%) of maternal and newborn QC samples, respectively. For the 162 metabolites examined here, the number of samples with detectable values in QC samples ranged from 63 to 300 (21.0 to 100%) with a mean of 247 (82.1%). Batch trends for a peak annotated ‘glucose and other aldohexoses’ and for 1,5-anhydroglucitol demonstrate the variability of batch effects for different metabolites. While metabolite levels tended to decrease for alanine from batches 28–45, levels for glucose increased across the same batches and levels for 1,5-anhydroglucitol jumped distinctly in the middle of this range at batch 35. Observations for all four of these metabolites illustrate run-order dependence. Abundance levels for QC samples at the beginning of each batch are frequently lower than abundance levels in the middle and at the end. Mixnorm adjusts for batch and run order for these four metabolites without requiring imputation for undetected values (Fig. 5, second column). Similar plots for these same four metabolites for the other approaches examined here are included in Additional file 6: Figures S10–S16. Of the methods applied, mean-centering, median scaling, quantile + ComBat, and Batch Normalizer accomplished similar stability of metabolite abundance across batches upon visual inspection for these four metabolites. Visual inspection also suggests that quantile normalization, EigenMS and VSN did not achieve comparable stability of QC data across batches.

**Fig. 6:**
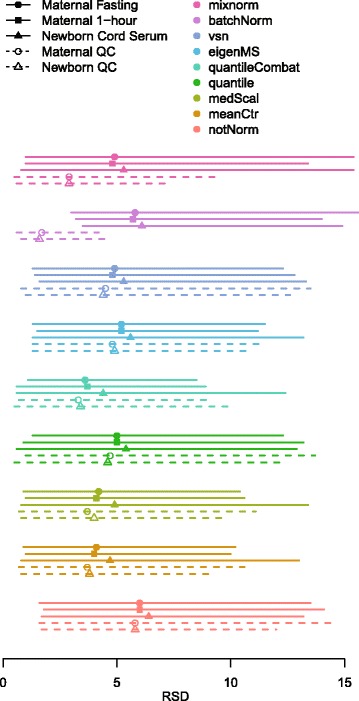
RSD values (%) for analytical (maternal fasting, maternal 1-hour, newborn cord serum) and QC (maternal QC, newborn QC) data in HAPO Metabolomics prior to and following normalization with each approach. Points correspond to the mean RSD and lines span the minimum to the maximum RSD for each sample type

Figure 6 plots means, minima and maxima for per-metabolite RSDs across the maternal and newborn QC samples and analytical samples (numeric results in Additional file 7). Compared to mean values of 5.8 for non-normalized maternal and newborn QC samples, substantial reductions in per-metabolite RSDs were evident in QC samples for quantile + ComBat (maternal QC mean 3.3, newborn QC mean 3.4), Batch Normalizer (maternal QC mean 1.7, newborn QC mean 1.6) and mixnorm (maternal and newborn QC means 2.9). Notably, quantile + ComBat resulted in similar per-metabolite variability for analytical samples as for QC samples. Effective control of technical variability should reduce per-metabolite RSDs for both QC and analytical samples; however, since QC samples are drawn from identical pools and in HAPO Metabolomics analytical samples are obtained from independent individuals from a population-based study, it is reasonable to expect that per-metabolite RSDs in QC samples would be substantially less than analytical samples. Batch Normalizer also yielded substantial reduction in per-metabolite RSDs in QC samples; however, per-metabolite RSDs for analytical samples remained almost identical to non-normalized data suggesting very little control of technical variability in samples of primary analytical interest. Consistent with the nature of HAPO Metabolomics study design, mixnorm reduces per-metabolite RSDs for maternal and newborn QC samples, with per-metabolite RSDs roughly 65–80% higher in analytical samples (means ranging 4.8–5.3 for the sample types). The remaining methods demonstrated less reduction in variability for QC samples than mixnorm, and were less reflective of higher per-metabolite variability expected for analytical compared to QC samples.

#### Pairwise correlations of QC samples

**Fig. 7:**
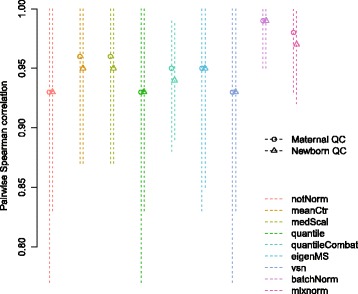
Pairwise Spearman correlation values for maternal and newborn QC samples in HAPO Metabolomics prior to and following normalization with each approach. Points correspond to the mean pairwise Spearman correlation value and lines span the minimum to the maximum pairwise Spearman correlation for each sample type. All Spearman correlation estimates are statistically significantly different from 0 with *p* < 0.05

Figure 7 illustrates the mean, minimum and maximum pairwise Spearman correlation coefficients of the maternal and newborn QC samples using data from metabolites with detected abundance and normalized values in at least 20% of samples (numeric results in Additional file 8). QC sample correlations were fairly high in non-normalized data with means of 0.93 for both maternal and newborn QCs. Compared to non-normalized data, pairwise correlations changed very little for quantile normalization and VSN and increased modestly for mean centering, median scaling, quantile + ComBat and EigenMS. Batch Normalizer and mixnorm increased pairwise correlations of QC samples the most with improvements in maternal QC samples to 0.99 and 0.98, respectively, and newborn QC samples to 0.99 and 0.97, respectively.

#### Correlations with conventional and targeted metabolites in analytical samples

**Fig. 8:**
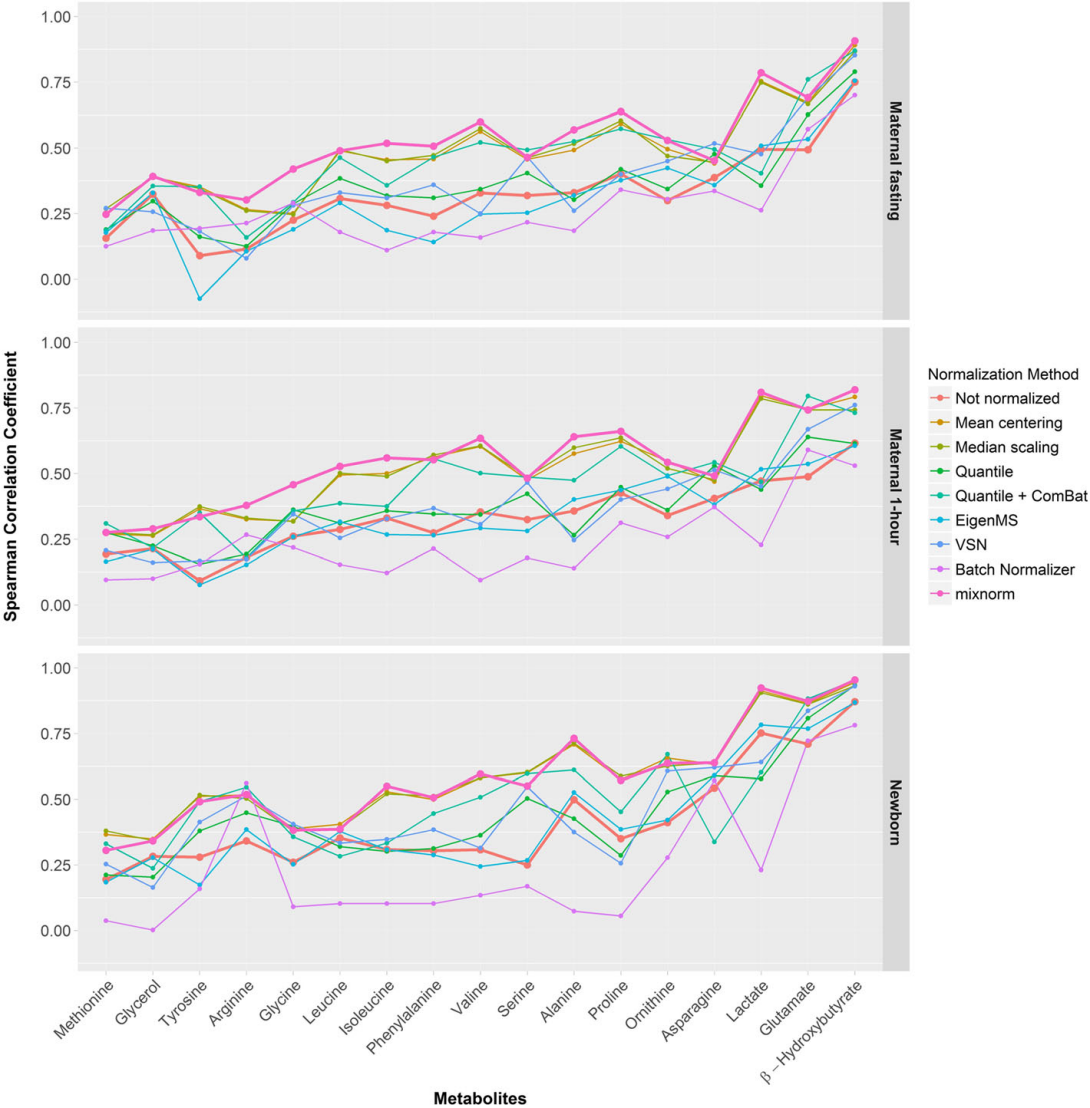
Spearman correlation coefficients for non-targeted and targeted data. Correlation estimates are plotted for non-targeted metabolites using each normalization method and their conventional metabolite or targeted amino acid counterparts. Results are presented separately for each analytical sample type. All Spearman correlation estimates are statistically significantly different from 0 with *p* < 0.05 with the exception of tyrosine after EigenMS normalization in maternal fasting samples and methionine, glycerol, alanine and proline after Batch Normalizer in cord serum samples

Conventional and targeted amino acid assays were used to detect the abundance of a subset of metabolites also detected by the non-targeted assays in HAPO Metabolomics. These data in some sense provide an external measure of non-targeted data normalization success since the conventional metabolite and targeted amino acid measurements are not subject to batch effects and do not require normalization. Analytical samples represent a full range of phenotypes and comparison of non-targeted to targeted data in these samples allows examination of the extent to which the normalization controls technical variability but preserves biologically relevant variability. Spearman correlation estimates in analytical samples are illustrated in Fig. 8, summarized in Table 2 and reported individually in Additional file 9. For these conventional / targeted analytes, the mean Spearman correlation estimates for the non-normalized data were 0.33, 0.33, and 0.41 for the maternal fasting, maternal 1-hour, and newborn cord serum samples, respectively. Quantile, EigenMS and VSN yielded correlations with targeted data that were comparable to values in non-normalized data. Batch Normalizer in general reduced correlations with targeted metabolites. While mean centering, median scaling, and quantile + ComBat yielded some improvement, mixnorm yielded the greatest gains in Spearman correlation among non-targeted metabolites and their targeted counterparts resulting in mean correlation estimates of 0.52, 0.54 and 0.59 for the maternal fasting, maternal 1-hour and newborn cord serum samples, respectively. Figure 8 illustrates the consistent increase in Spearman correlation for mixnorm across all represented conventional metabolites and targeted amino acids in all analytical sample types.

**Table 2:** Summary statistics for Spearman correlation coefficients between non-targeted and targeted assays

	Spearman correlation estimate summary statistics: mean (min, max)		
	Maternal fasting	Maternal 1-hour	Newborn cord serum
Not normalized	.33 (.09, .75)	.33 (.09, .62)	.41 (.19, .87)
Mean centering	.49 (.24, .89)	.51 (.26, .80)	.59 (.35, .94)
Median scaling	.49 (.25, .87)	.51 (.27, .79)	.59 (.34, .93)
Quantile	.36 (.13, .79)	.37 (.15, .64)	.45 (.20, .93)
Quantile + ComBat	.46 (.16, .87)	.46 (.18, .80)	.51 (.24, .95)
EigenMS	.30 (−.07, .75)	.33 (.08, .61)	.42 (.17, .87)
VSN	.38 (.08, .85)	.37 (.16, .76)	.47 (.16, .93)
Batch normalizer	.27 (.11, .70)	.24 (.10, .59)	.25 (.00, .78)
mixnorm	.52 (.25, .91)	.54 (.28, .82)	.59 (.31, .95)

#### Associations with HAPO phenotypes

**Fig. 9:**
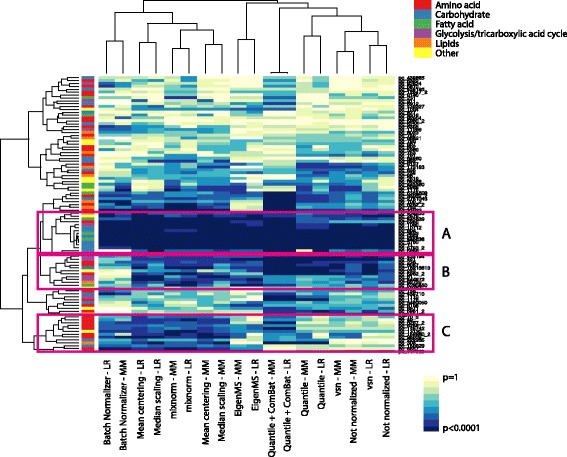
Heatmap of associations with maternal fasting plasma glucose (FPG) for fasting maternal metabolites in HAPO Metabolomics. The colors on the heatmap correspond to the strength of association with dark blue representing p-values close to 0 and light yellow representing *p*-values close to 1. Associations were detected using both linear regression and downstream mixture modeling prior to and following normalization with each approach. Hierarchical clustering was applied to columns and rows. Columns are close to each other for methods that detect similar associations. Rows are close each other if the strength of detected associations for the metabolites (represented by PubChem ID starting with ‘pc_’) are similar across the range of methods. Compound classes for each metabolite are represented by the lefthand vertical bar (*red* – amino acids; *blue* – carbohydrates; *green* – fatty acids; *purple* – glycolysis/tricarboxylic acid cycle; *orange* – lipids; *yellow* – other). *Pink* boxes A, B and C highlight clusters of metabolites detected by different sets of normalization approaches

Similar to simulated data, associations of fasting maternal metabolite levels with maternal FPG in HAPO data were identified using both linear regression and downstream mixture modeling after application of the normalization methods. Figure 9 is a heatmap with hierarchical clustering for both rows and columns illustrating results of per-metabolite associations detected for each method, with dark blue shading corresponding to lower *p*-values and light yellow to higher p-values. A cluster of significant metabolite associations with FPG largely comprised of carbohydrates, indicated by the pink highlighted box A, is detected after application of all normalization methods. The cluster identified by pink highlighted box B contains primarily a mix of amino acids and glycolysis/tricarboxylic acid metabolites, and the cluster identified by pink highlighted box C contains primarily amino acids. Association analyses after mixnorm, mean centering and median scaling normalization leads to the identification of significant FPG associations within these compound classes, all of which are known to be associated with pregnancy-related maternal glycemia [40, 41]. Pathway analyses confirm that these three methods also lead to the highest number of significantly enriched pathways that involve these same compound classes (Additional file 10).

## Discussion

We propose a mixture-model normalization approach for GC/MS non-targeted metabolomics data called mixnorm that estimates metabolite-specific batch and run order effects based on QC samples. Mixnorm easily accommodates multiple QC sample types, an important feature for experiments that include samples from different sources, different types of individuals, etc. Additionally, rather than ignoring undetected metabolites or relying on imputation of their values, mixnorm formally models detectability or lack thereof for low abundance metabolites and accommodates batch-specific detectability thresholds. This is a more precise handling of truncated data than simple imputation of a low-valued constant or reliance on algorithmic approaches that often impute values in the range of observed values thus inconsistent with the notion of low abundance. Given the specific corrections estimated for each metabolite, mixnorm is applicable for the full set of mass spectrometry peaks following decomposition or for a desirable subset, for example in our case the peaks that were reliably annotated in the AMDIS-based pipeline we applied.

In simulations, when compared to other methods, mixnorm demonstrated the most accurate recovery of true RSDs for both QC and analytical sample data, even in the presence of substantial proportions of undetected values due to data truncation. Mixnorm also yielded a very high true positive probability for detecting associations with the simulated phenotype, with only mean centering and median scaling showing comparable performance on this particular metric. In analyses of HAPO Metabolomics data, mixnorm demonstrated reduction in RSD with patterns that most reasonably reflect expected lower RSD values for QC data compared to RSD values for analytical data. Mixnorm also demonstrated consistent improvement in pairwise Spearman correlation coefficients among QC samples. Importantly, when compared to targeted measurements of the same metabolites in HAPO Metabolomics samples, mixnorm yielded the highest and most consistent improvement in Spearman correlation coefficients across all methods. Phenotype association analyses and pathway analyses using HAPO Metabolomics data also confirm the ability of mixnorm to detect meaningful associations of biological relevance.

Normalization is just one component of carefully crafted pipelines that should be applied to perform high quality metabolomics experiments. Rigorous protocols for sample collection and storage, compound derivatization and metabolite extraction, and reproducible compound annotation pipelines are paramount to successful study conduct [2–5]. Normalization procedures that rely on QC data should take note of potential outlying observations. Summaries of HAPO Metabolomics data indicated that none of the QC observations fell outside 3 standard deviations within a given metabolite; hence, we determined that all QC observations could be used for parameter estimation. We do recommend that investigators identify potential outlying QCs; the *mixnorm* function in the *metabomxtr* R package supports outlier filtering. It is also recommended that investigators take note of potential outliers in analytical samples that may influence observed phenotype associations even after data normalization is performed.

Effective normalization using QC controls also requires careful attention to experimental design. The HAPO Metabolomics study was designed to examine metabolic profiles in mothers at fasting and 1-hour into an OGTT and in their newborns’ cord serum. We randomly sampled mother/baby sample triples for placement into batches to balance continuous traits of interest across batches to the extent possible. Given expected differences in maternal and newborn metabolic profiles, we created separate QC pools to resemble the full retention time distribution of each sample type, and strategically placed QC samples from each pool at the beginning, middle and end of each batch to capture run order effects. We do note that the HAPO Metabolomics Study utilized stored samples and thus common QC pools could be created at the outset of the experiment for all batches. In ongoing studies for which samples accumulate over time, investigators may need to utilize QC pools from external sources or build adequate sized pools from initial samples that can be utilized over the anticipated duration of the study. HAPO Metabolomics also included two types of QC pools to mirror the maternal and newborn sample types involved in the study. We note that mixnorm was applied to all QC data simultaneously, with a covariate for QC sample type included in the mixture model along with batch and run order covariates. Hence, batch and run order effects were estimated using data from all QCs and location shifts were uniformly applied to all maternal and newborn analytical samples. This is an important, albeit subtle, point especially for studies in which sample classification is the goal. Analytical sample types may not be known a priori, but if QC pools can be obtained that are similar in nature to the anticipated classes, batch and run order effects can be estimated using multiple QC types and location shifts applied to all analytical samples in the same way we used mixnorm for HAPO Metabolomics data. Additionally, we note that three QC samples of a given type placed evenly within each batch produced reasonably stable parameter estimates for batches including 24 analytical samples in both the simulation and HAPO Metabolomics. Fewer QC samples would likely lead to less precise effect estimates. The ability to invest in QCs is likely to vary substantially from study to study; if possible, it may be useful to conduct preliminary studies including QC samples to estimate reasonable QC sample sizes for stable parameter estimation. Exact experimental design specifications will depend on the study, but classic principles of covariate balance, sample matching, and thoughtful QC sample creation and placement should be strongly considered when designing batches for large-scale metabolomics experiments.

This investigation describes the use of mixture modeling for normalization purposes. As discussed in phenotypic association analyses for both the simulated data and the HAPO Metabolomics GC/MS data, mixture modeling can also be applied for downstream analyses with covariates specified to represent variables of biological, epidemiological and/or clinical interest. The main emphasis of this manuscript is the utility of the mixture model for control of technical noise related to batch and run order in large-scale GC/MS studies, but the general modeling strategy has other uses as well.

## Conclusion

In summary, we propose mixnorm for normalization of data from large-scale non-targeted GC/MS metabolomics studies. While application of the method requires use of multiple QC samples from one or more control pools over the course of the experiment, these control pools can typically be generated using small extractions from the samples of analytical interest without compromising the integrity of analytical samples for non-targeted profiling. Simulation studies confirm that mixnorm accommodates a far higher proportion of undetected metabolite values while maintaining more accurate estimates of RSD than other methods evaluated here. This is crucial for accurately modeling and analyzing low abundance compounds that may be subject to batch-specific truncation. Across global metrics including metabolite RSDs and pairwise correlations for QC samples, mixnorm showed consistent and marked improvement using data from the HAPO Metabolomics study case study. When evaluated with reference to conventional and targeted assays of a subset of metabolites reflecting a range of phenotypes in HAPO analytic samples of interest, mixnorm, along with mean centering and median scaling, accomplished the greatest increases in Spearman correlations compared to the other methods. Both simulation results and the case study using HAPO Metabolomics data also indicate reliable detection of phenotypic associations when GC/MS data are normalized using mixnorm, with comparable performance by mean centering and median scaling. It is possible that results may vary depending on phenotypes of interest in other metabolomics studies. Mixnorm can be implemented using functionality in the metabomxtr R package (devel version) [23] available at http://www.bioconductor.org/ [26].

## Additional files

Additional file 1: Extended descriptions of normalization approaches including software, and a table comparing features of the methods. (DOCX 93 kb)
Additional file 2: R functions for mean centering, median scaling and Batch Normalizer that are not available in the R and Bioconductor packages used for the other normalization methods used in this study. (ZIP 12 kb)
Additional file 3: HAPO Metabolomics data used for analysis. (XLSX 2773 kb)
Additional file 4: Figures S1–S9. Plots of RSD for simulated QC and analytical samples prior to and following normalization for randomly selected simulation rounds. **Figure S1.** RSD plots for simulation round 101. **Figure S2.** RSD plots for simulation round 115. **Figure S3.** RSD plots for simulation round 123. **Figure S4.** RSD plots for simulation round 190. **Figure S5.** RSD plots for simulation round 583. **Figure S6.** RSD plots for simulation round 732. **Figure S7.** RSD plots for simulation round 826. **Figure S8.** RSD plots for simulation round 866. **Figure S9.** RSD plots for simulation round 880. (PDF 14641 kb)
Additional file 5: True positive and false positive probabilities of detecting metabolite associations of varying strengths with a simulated phenotype. (DOCX 101 kb)
Additional file 6: Figures S10–S16. Plots of maternal and newborn QC HAPO Metabolomics samples prior to and following normalization for four selected metabolites. **Figure S10.** Plots of QC samples prior to and following mean centering. **Figure S11.** Plots of QC samples prior to and following median scaling. **Figure S12.** Plots of QC samples prior to and following quantile normalization. **Figure S13.** Plots of QC samples prior to and following quantile + ComBat. **Figure S14.** Plots of QC samples prior to and following EigenMS. **Figure S15.** Plots of QC samples prior to and following VSN. **Figure S16.** Plots of QC samples prior to and following Batch Normalizer. (PDF 774 kb)
Additional file 7: Summary statistics for RSD for HAPO Metabolomics samples according to sample type prior to and following normalization. Lower RSD indicates better performance of the normalization method. RSD values for QC samples are expected to be lower than RSD for analytical samples since QCs are from common pools. (DOCX 75 kb)
Additional file 8: Summary statistics for pairwise Spearman correlation among HAPO Metabolomics QC samples prior to and following normalization. Higher pairwise Spearman correlation indicates better performance of the normalization method. (DOCX 51 kb)
Additional file 9: Spearman correlation coefficients for non-targeted metabolites with conventional metabolites and targeted amino acid data in HAPO Metabolomics. (DOCX 23 kb)
Additional file 10: MetaboAnalyst 3.0 pathway results for each normalization method and both linear regression and mixture model analysis of phenotypic associations in HAPO Metabolomics. (DOCX 140 kb)

## Abbreviations

GC/MS: Gas chromatography/Mass spectrometry
HAPO: Hyperglycemia and Adverse Pregnancy Outcome
OGTT: Oral glucose tolerance test
QC: Quality control
RSD: Relative standard deviation
RTL: Retention-time-lock
VSN: Variance stabilizing normalization
