# Supplementary material for: Mixture model normalization for non-targeted gas chromatography/mass spectrometry metabolomics data
Source: BMC Bioinformatics. 2017 Feb 2;18:84. doi: 10.1186/s12859-017-1501-7 (PMC5290663; doi:10.1186/s12859-017-1501-7)
Supplement: Additional file 1: — Extended descriptions of normalization approaches including software, and a table comparing features of the methods. (DOCX 93 kb) [file 12859_2017_1501_MOESM1_ESM.docx]

**Additional File 1:** Feature comparisons for normalization methods. Extended descriptions for methods other than mixnorm are included below the table. Mixnorm is described in the main text.

| **Method** | **QC samples** | **Batch effects** | **Run order effects** | **Metabolite-specific or global adjustment** | **Undetected metabolites** |
| --- | --- | --- | --- | --- | --- |
| Mean centering | No use of QC samples. | Metabolite expression values are centered around metabolite- and batch-specific means. | Ignores run-order effects. | Metabolite-specific. | Ignores missing data/truncation. |
| Median scaling | No use of QC samples. | Metabolite expression values are divided by their batch-specific medians. | Ignores run-order effects. | Metabolite-specific. | Ignores missing data/truncation. |
| Quantile Normalization | No use of QC samples. | Does not explicitly model batch effects. | Ignores run-order effects. | Global adjustment. | Ignores missing data/truncation. |
| Quantile + ComBat | No use of QC samples. | Corrects for batch effects by invoking prior distributions with empirically estimated parameters. | Ignores run-order effects. | Global adjustment. | Cannot perform if a batch-phenotype combination for any metabolite is missing. |
| EigenMS | No use of QC samples. | Does not explicitly model batch effects, but removes bias trends in the data that may arise from batch variability. | Ignores run-order effects. | Global adjustment. | Ignores missing data/truncation. |
| Batch Normalizer | Uses QC samples to estimate corrections for batch and run order effects. No formal method for including multiple QC sample types. | Corrects for batch effects by using a regression algorithm on QC samples to estimate corrections for batch effects that are then applied to analytical samples. | Corrects for run-order effects by using a regression algorithm on QC samples to estimate corrections for run order that are then applied to analytical samples. | Metabolite-specific. | Ignores missing data/truncation. |
| VSN | No use of QC samples. | Does not explicitly model batch effects, but removes bias trends in the data that may arise from batch variability. | Ignores run-order effects. | Global adjustment. | Ignores missing data/truncation. |
| mixnorm | Uses QC samples to estimate corrections for batch and run order effects. Multiple QC types easily accommodated by including a QC sample type covariate in the mixture model. | Corrects for batch effects by using a mixture model regression algorithm on QC samples to estimate corrections for batch effects that are then applied to analytical samples. | Corrects for run-order effects by using a mixture model regression algorithm on QC samples to estimate corrections for run order that are then applied to analytical samples. | Metabolite-specific. | Models the detectability or lack thereof of a given metabolite in a sample and allows specification of batch-specific thresholds of detectability. |

***Mean Centering*** Mean centering was originally used for combining gene expression microarray data from different platforms [1]. In our application, for each metabolite in a given sample, the mean of the metabolite level across all samples in the same batch was subtracted from the observed metabolite level. Since this first step results in a mean of each mean-centered metabolite equal to zero, we then added a constant to each metabolite level equal to the mean of that same metabolite across all samples in all batches so that data values resembled the original scale. ***Software:*** In house programming was used to perform mean centering. Code is available from the authors upon request.

***Median Scaling*** Median scaling employs a scaling factor based on metabolite medians [2]. While there are various approaches to median scaling, for our application, each metabolite abundance level in a given batch was divided by the ratio of its batch-specific median to the median for that metabolite across all samples in all batches. ***Software:*** In house programming was used to perform median scaling. Code is available from the authors upon request.

***Quantile Normalization*** Quantile normalization aims to make the distribution of metabolite abundance levels the same across all samples [3]. This is accomplished by sorting the metabolite abundance levels for each sample, taking the means of the ranked metabolites across all samples, and substituting these mean values for the original metabolite levels such that the highest value in all samples becomes the mean of the highest values, the second highest value becomes the mean of the second highest values, and so on. ***Software:*** Quantile normalization was implemented using the preprocessCore (version 1.36.0) R package, available at [www.bioconductor.org](http://www.bioconductor.org) [4].

***Quantile + ComBat*** Quantile normalization, as described above, was performed and followed by ComBat. ComBat is an empirical Bayes method using metabolite-specific estimations of mean and variance to correct for batch effects, while maintaining treatment or phenotype effects. It is intended to be applied in conjunction with other normalization algorithms, specifically for batches with small sample size (<25) [5]. ComBat cannot perform if metabolite data for a batch-phenotype combination are completely missing. Due to this occurrence in our data, missing values were imputed prior to Quantile + ComBat using Bayesian PC analysis. In instances when this imputation method resulted in negative values, the half-minimum value from the pre-imputed metabolite was substituted. ***Software:*** Bayesian PC analysis was performed using method from the pcaMethods (version 1.66.0) R package. Quantile + ComBat were implemented using the preprocessCore (version 1.36.0) and sva (version 3.22.0) R packages. The pcaMethods, preprocessCore, and sva R packages are available at [www.bioconductor.org](http://www.bioconductor.org) [4].

***EigenMS*** EigenMS is based on surrogate value analysis originally proposed for microarray gene expression data and adapted for non-targeted metabolomics data [6, 7]. The algorithm first requires estimation of a categorical ‘treatment’ effect via ANOVA; in our application we specified 5 categories corresponding to the 5 sample types (maternal QC, fasting and 1-hour samples and newborn QC and cord serum samples). Singular value decomposition is then applied to the matrix of residuals and additional bias trends are removed from the data for a final normalized data set. ***Software:*** EigenMS was implemented in our application using the EigenMS R functions, available at [www.sourceforge.net](http://www.sourceforge.net).

***Batch Normalizer*** Batch Normalizer is a regression-based algorithm that incorporates total abundance of each sample when estimating corrections for batch and run order effects [8]. First the median metabolite abundance level across all metabolites in all control and analytical samples is observed. Then, all metabolite levels are scaled by the ratio of this median to the sum of all observed metabolite levels for the sample (either control or analytical sample of interest) to which it belongs. Using control samples only, a linear regression model on the scaled metabolite abundances is then used to estimate a batch-specific intercept and run order effects assuming linearity. The scaled metabolite abundance levels in the analytical samples of interest are then rescaled by the ratio of the median abundance of the metabolite of interest in the control samples to the sum of the batch-specific intercept and estimated run order effect for that sample’s run order position. ***Software:*** Batch Normalizer was implemented in our application using in-house programming of the published algorithm. Code is available from the authors upon request.

***Variance Stabilizing Normalization (VSN)*** VSN was originally developed for gene expression microarray data normalization and accounts for the fact that variance of observed gene expression values depends on signal intensity [9]. VSN applies a smooth transformation to all expression values that mimics a log transformation for high intensity values and linear scaling for low intensity values, rendering variance approximately constant across the full range of intensities. VSN assumes that at least half of all values being normalized are constant across experimental conditions or phenotypes. ***Software:***  VSN was performed using the vsn (version 3.42.3) R package, available at [www.bioconductor.org](http://www.bioconductor.org) [4].

References

1. Sims AH, Smethurst GJ, Hey Y, Okoniewski MJ, Pepper SD, A. H, Miller CJ, Clarke RB: **The removal of multiplicative, systematic bias allows integration of breast cancer gene expression datasets - improving meta-analysis and prediction of prognosis**. *BMC Medical Genomics* 2008, **1**:42.

2. Wang W, Zhou H, Lin H, Roy S, Shaler TA, Hill LR, Norton S, Kumar P, Anderle M, Becker CH: **Quantification of proteins and metabolites by mass spectrometry without isotopic labeling or spiked standards**. *Analytical Chemistry* 2003, **75**:4818-4826.

3. Bolstad BM, Irizarry RA, Astrand M, Speed TP: **A comparison of normalization methods for high density oligonucleotide array data based on variance and bias**. *Bioinformatics* 2003, **19**(2):185-193.

4. Gentleman RC, Carey VC, Bates DM, Bolstad BM, Dettling M, Dudoit S, Ellis B, Gautier L, Ge Y, Gentry J *et al*: **Bioconductor: open software development for computational biology and bioinformatics**. *Genome Biology* 2004, **5**:R80.

5. Johnson WE, Li C, Rabinovic A: **Adjusting batch effects in microarray expression data using empircal Bayes methods**. *Biostatistics* 2007, **8**(1):118-127.

6. Leek JT, Storey JD: **Capturing heterogeneity in gene expression studies by surrogate variable analysis**. *PLoS Genetics* 2007, **3**(9):e161.

7. Karpievitch YV, Nikolic SB, Wilson R, Sharman JE, Edwards LM: **Metabolomics data normalization with EigenMS**. *PLOS One* 2014, **9**(12):e116221.

8. Wang S-Y, Kuo C-H, Tseng YJ: **Batch Normalizer: A Fast Total Abundance Regression Calibration Method to Simultaneously Adjust Batch and Injection Order Effects in Liquid Chromatography/Time-of-Flight Mass Spectrometry-Based Metabolomics Data and Comparison with Current Calibration Methods**. *Analytical Chemistry* 2013, **85**:1037-1046.

9. Huber W, von Heydebreck A, Sültmann H, Poustka A, Vingron M: **Variance stabilization applies to microarray data calibration and to the quantification of differential expression**. *Bioinformatics* 2002, **18 suppl 1**:S96-S104.
