# Supplementary material for: Mixture model normalization for non-targeted gas chromatography/mass spectrometry metabolomics data
Source: BMC Bioinformatics. 2017 Feb 2;18:84. doi: 10.1186/s12859-017-1501-7 (PMC5290663; doi:10.1186/s12859-017-1501-7)

Figure S1: Simulation 101

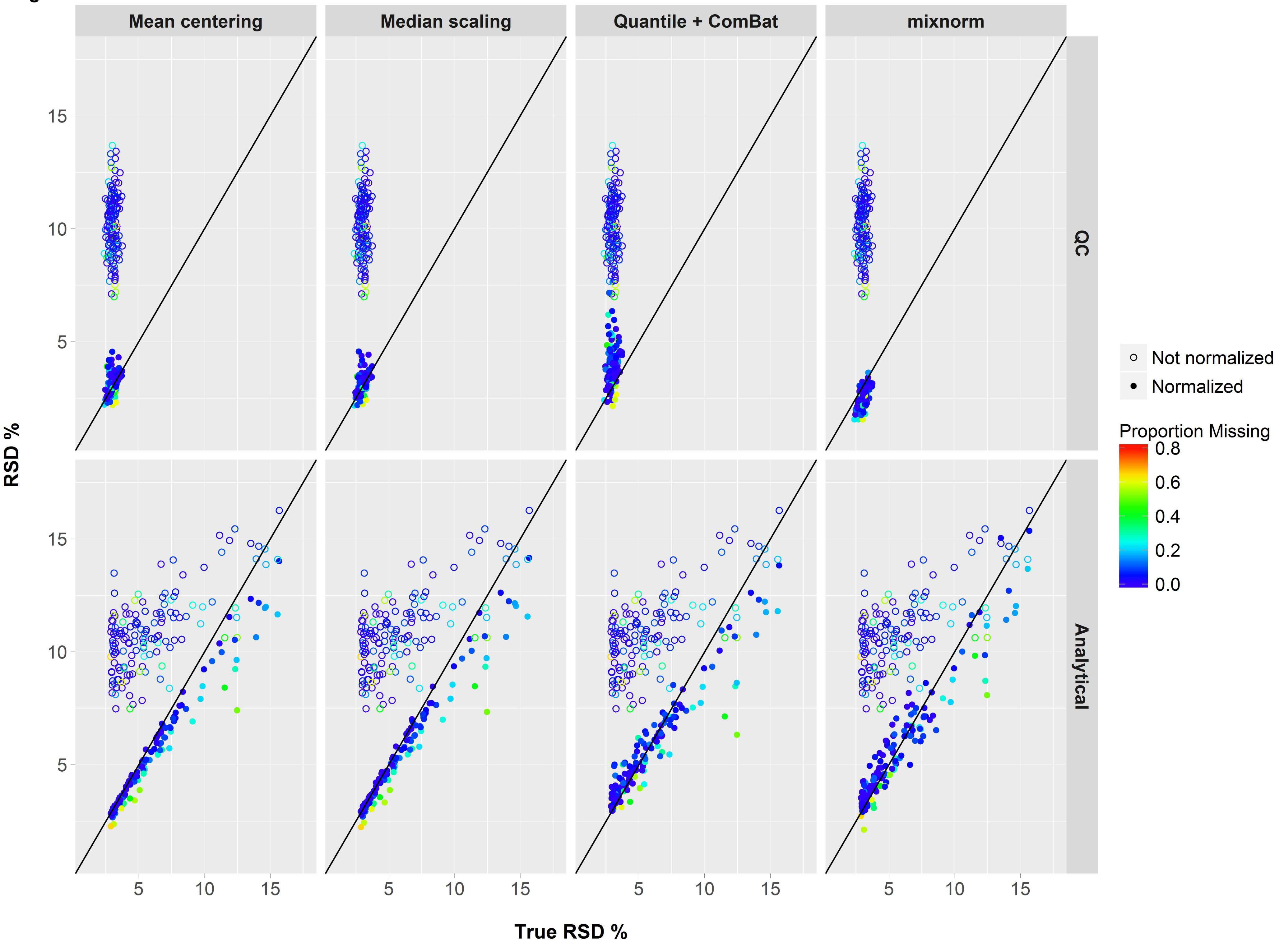

Figure S2: Simulation 115

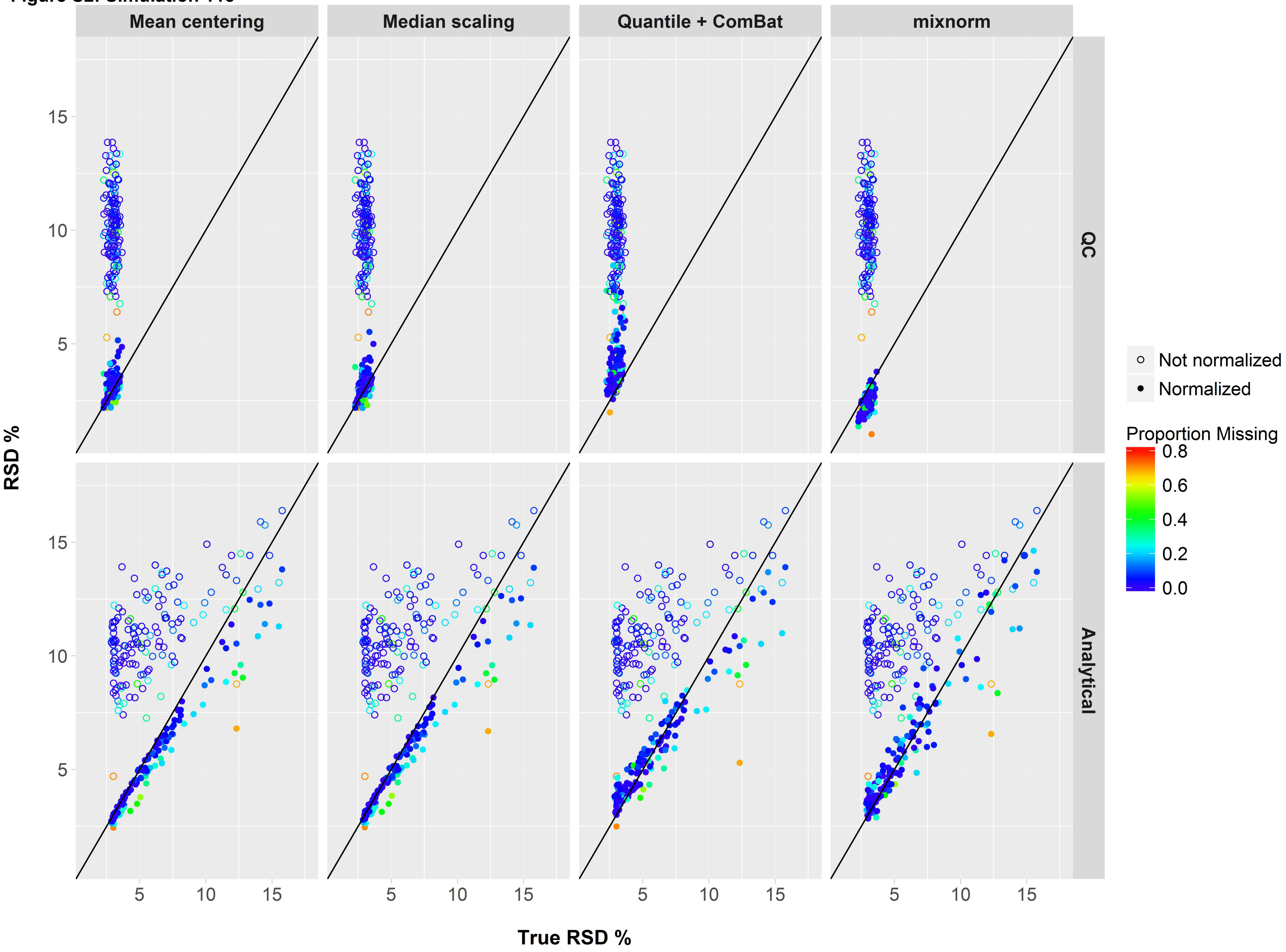

Figure S3: Simulation 123

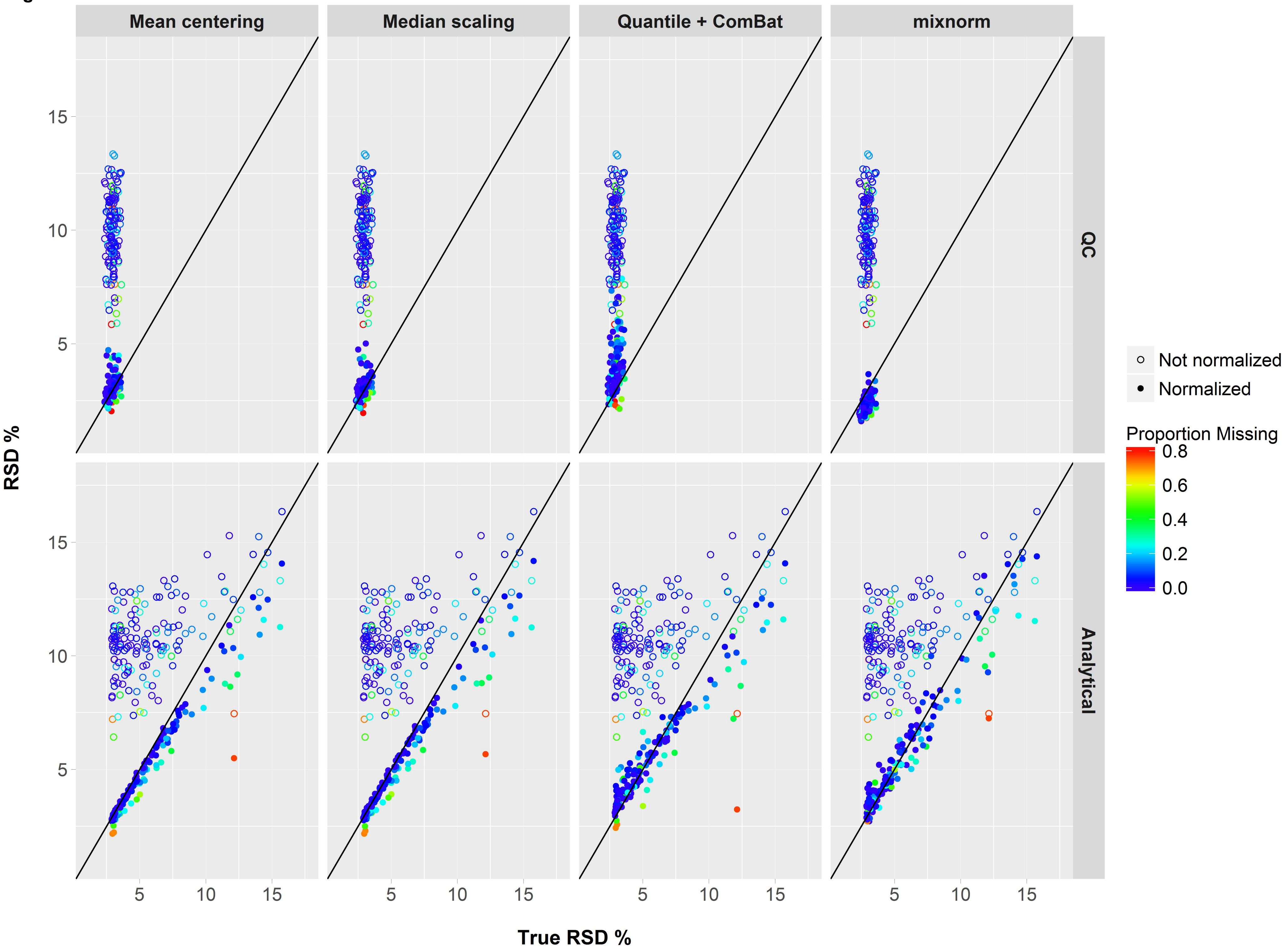

Figure S4: Simulation 190

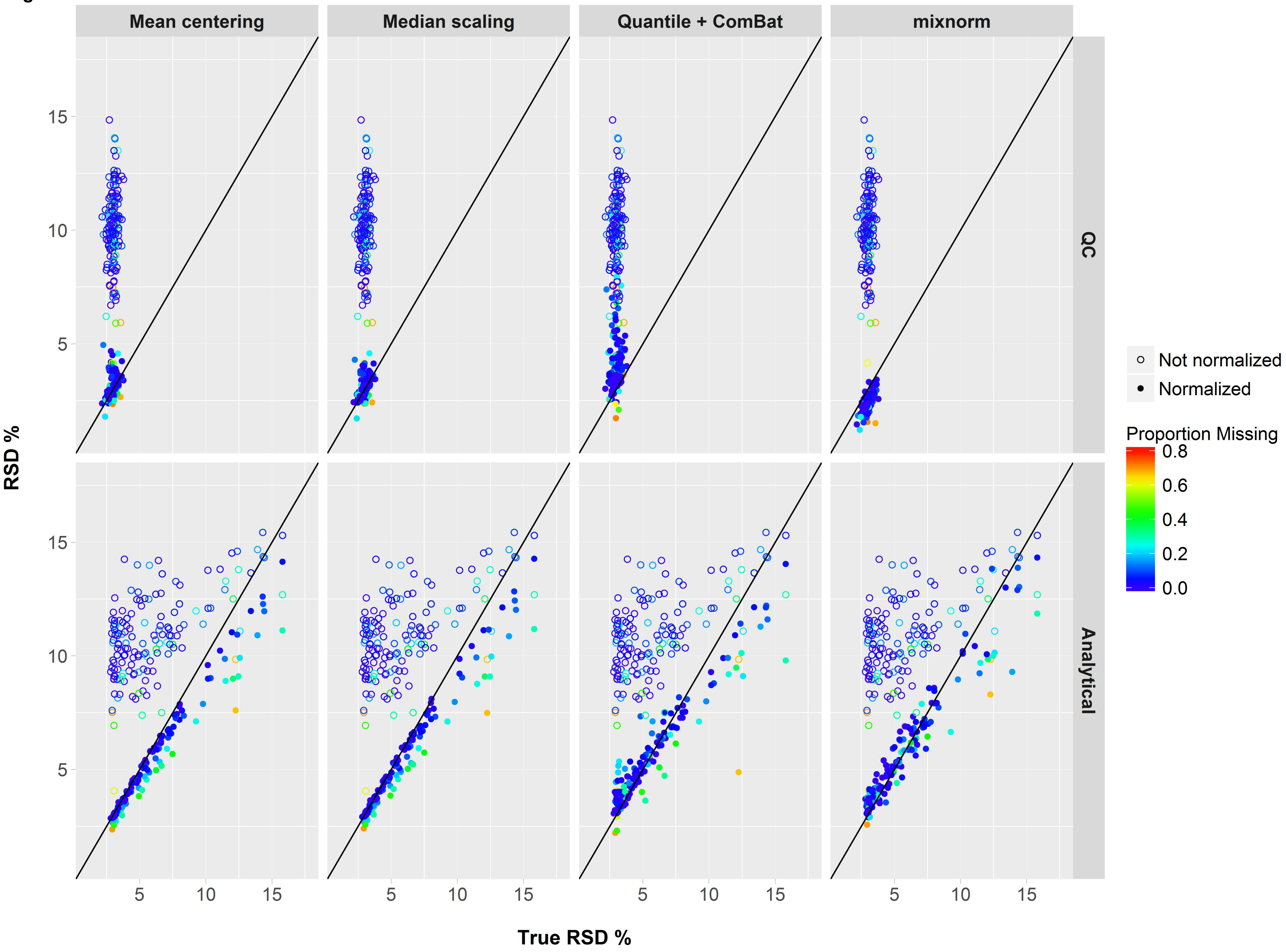

Figure S5: Simulation 583

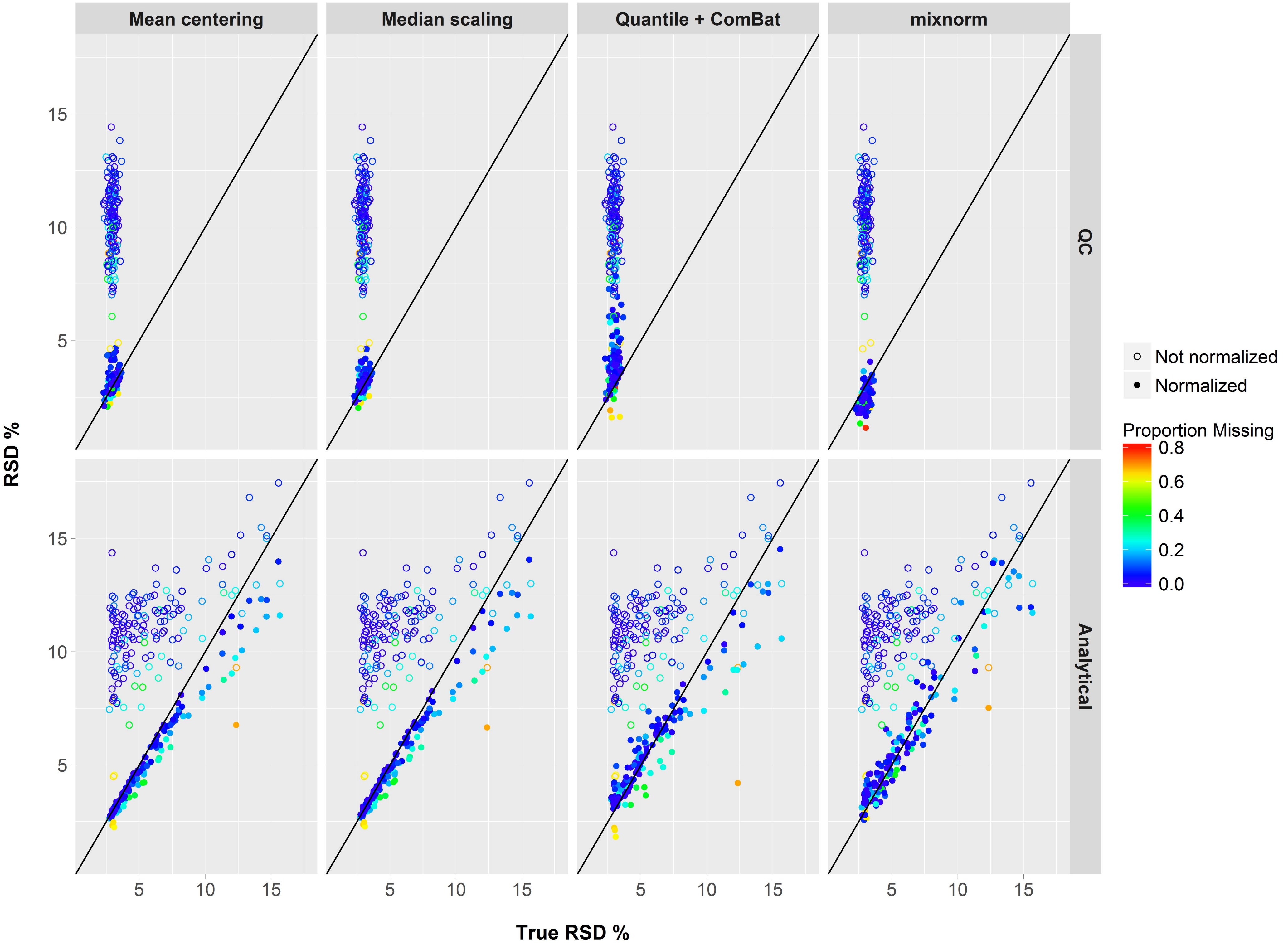

Figure S6: Simulation 732

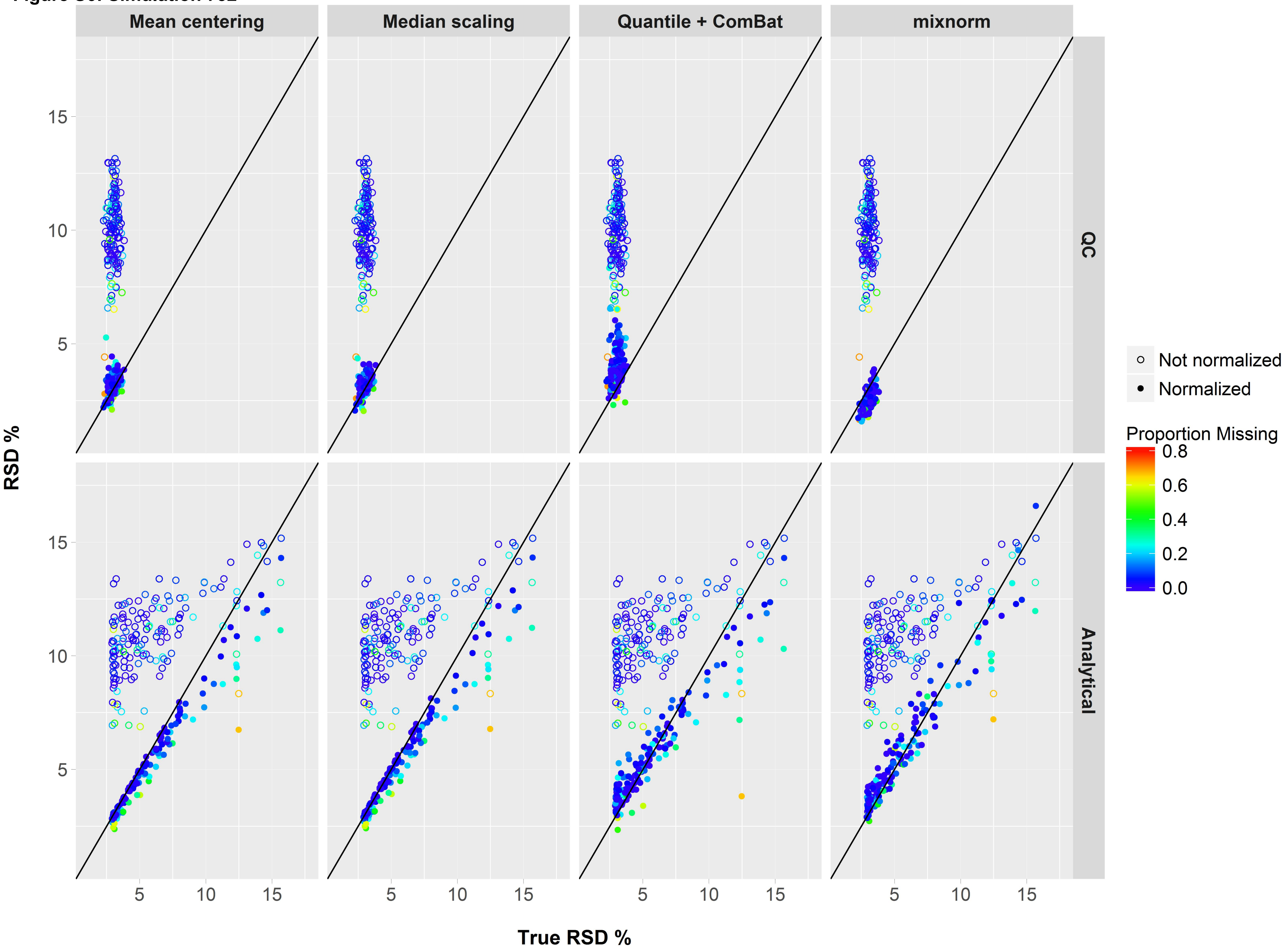

Figure S7: Simulation 826

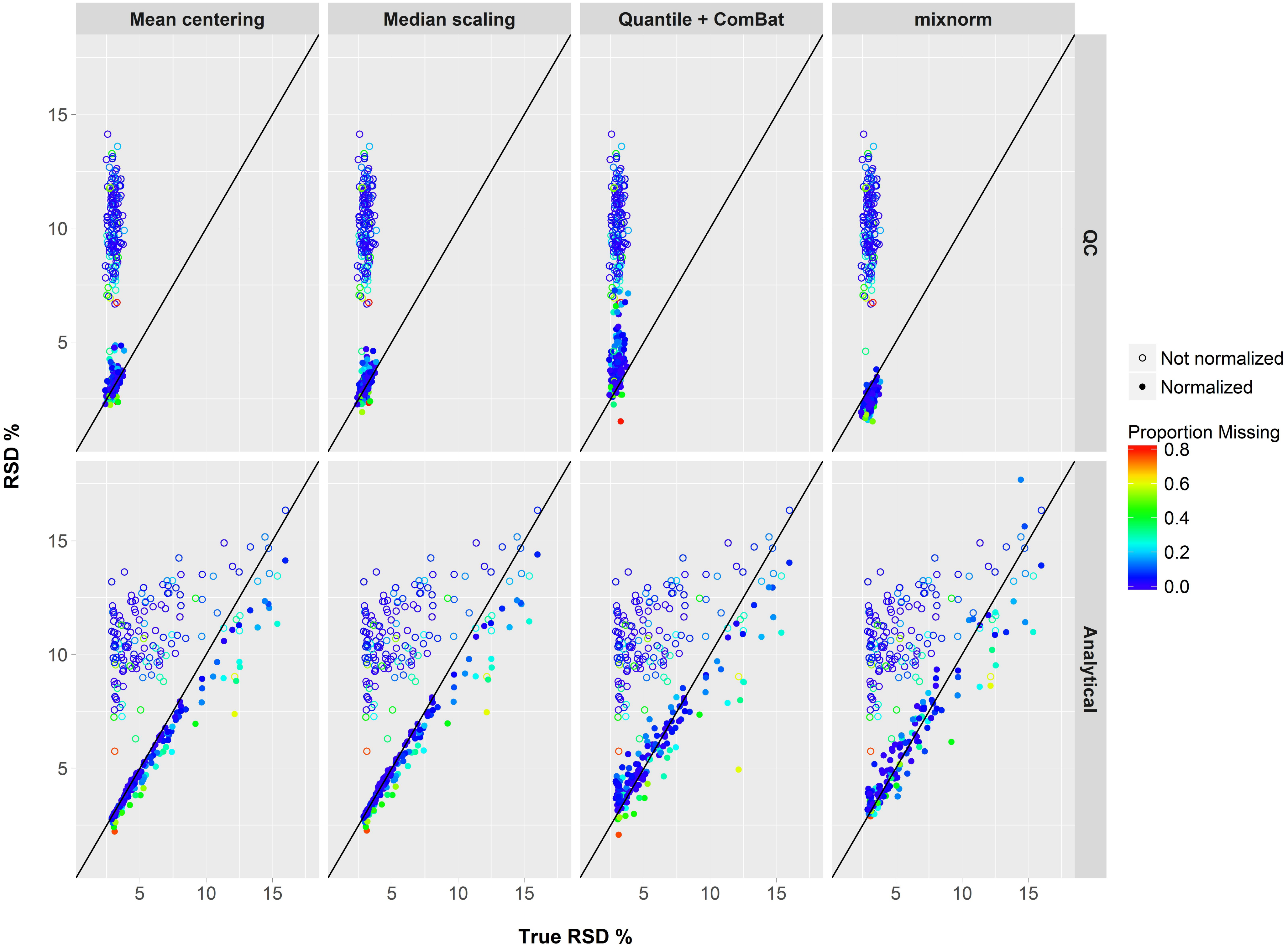

Figure S8: Simulation 866

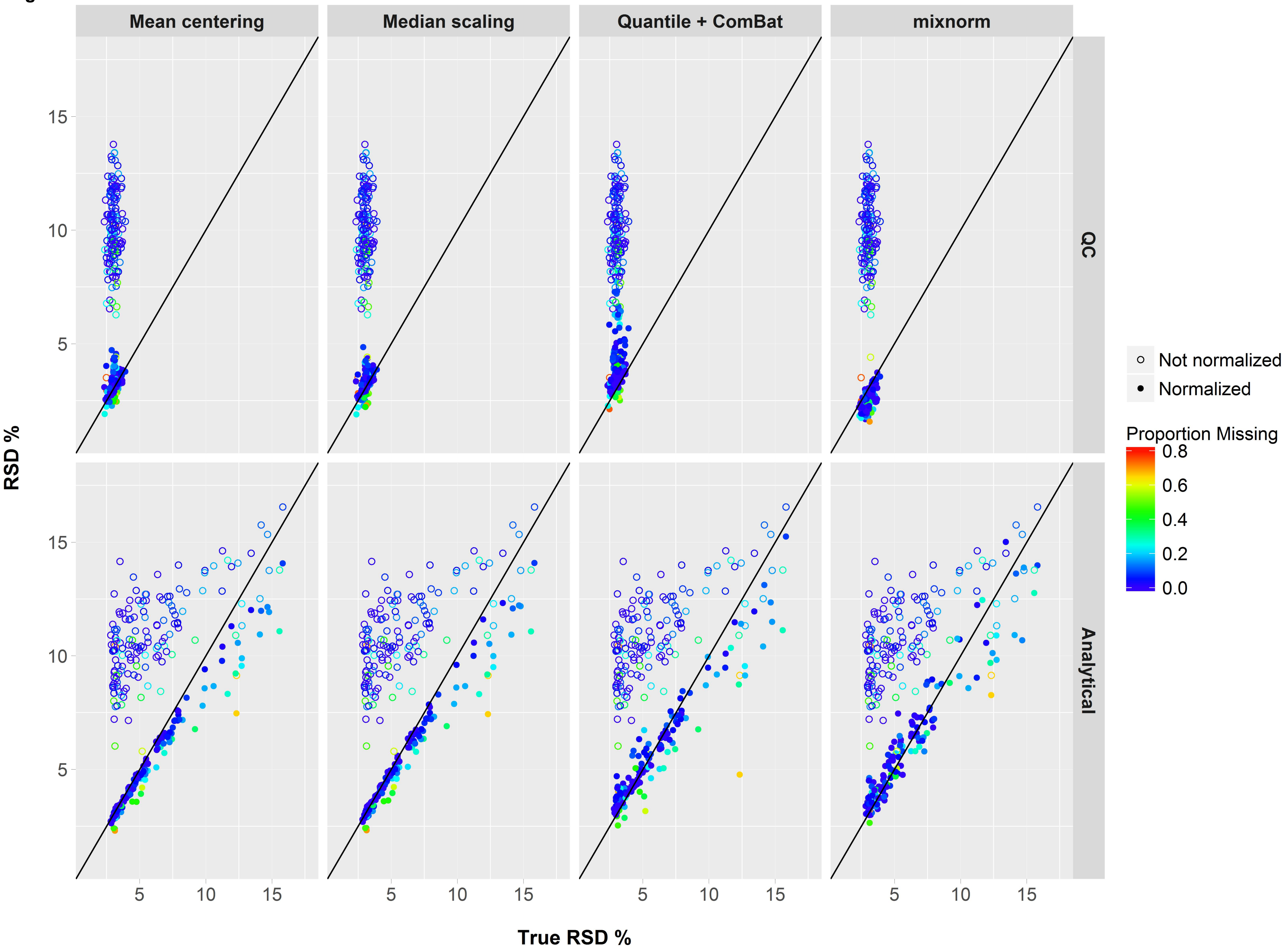

Figure S9: Simulation 880

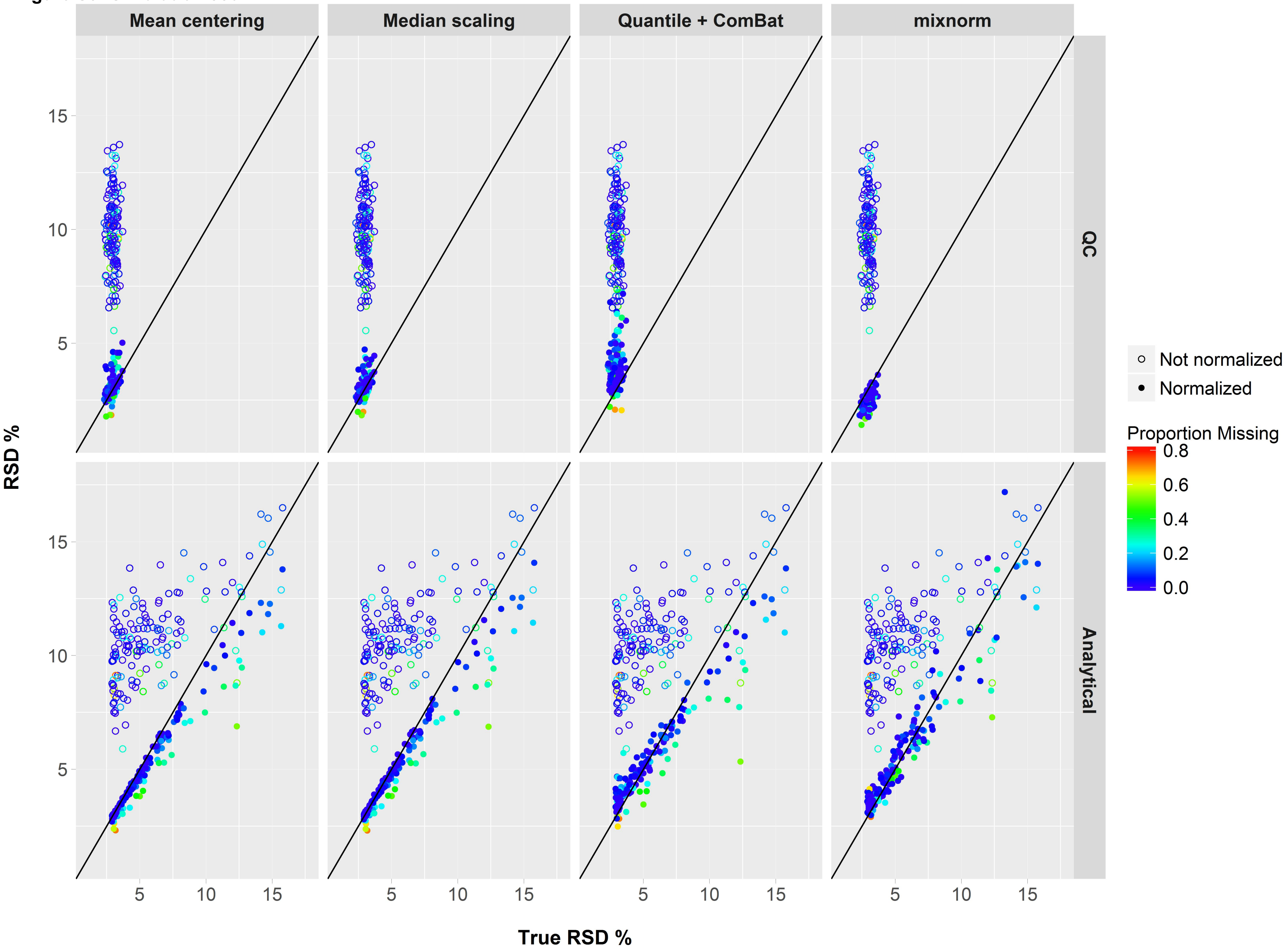

Supplement: Additional file 4: Figures S1–S9. — Plots of RSD for simulated QC and analytical samples prior to and following normalization for randomly selected simulation rounds. Figure S1. RSD plots for simulation round 101. Figure S2. RSD plots for simulation round 115. Figure S3. RSD plots for simulation round 123. Figure S4. RSD plots for simulation round 190. Figure S5. RSD plots for simulation round 583. Figure S6. RSD plots for simulation round 732. Figure S7. RSD plots for simulation round 826. Figure S8. RSD plots for simulation round 866. Figure S9. RSD plots for simulation round 880. (PDF 14641 kb) [file 12859_2017_1501_MOESM4_ESM.pdf]
