# Supplementary material for: Mixture model normalization for non-targeted gas chromatography/mass spectrometry metabolomics data
Source: BMC Bioinformatics. 2017 Feb 2;18:84. doi: 10.1186/s12859-017-1501-7 (PMC5290663; doi:10.1186/s12859-017-1501-7)
Supplement: Additional file 5: — True positive and false positive probabilities of detecting metabolite associations of varying strengths with a simulated phenotype. (DOCX 101 kb) [file 12859_2017_1501_MOESM5_ESM.docx]

| **Additional File 3:** True positive and false positive probabilities for detecting associations with a simulated phenotype after application of each normalization method | | | | | | | | |
| --- | --- | --- | --- | --- | --- | --- | --- | --- |
| Linear regression | True positive probabilities  for betas with  absolute value ≥ | | | | | | | False positive probability  (betas with absolute value ≤0.01) |
|  | 0.05 | 0.1 | 0.2 | 0.3 | 0.4 | 0.5 | 1.0 |  |
| Not normalized | .86 | .90 | .95 | .97 | .99 | .99 | 1 | .07 |
| Mean centering | .99 | 1 | 1 | 1 | 1 | 1 | 1 | .06 |
| Median scaling | .99 | 1 | 1 | 1 | 1 | 1 | 1 | .06 |
| Quantile | .84 | .88 | .94 | .96 | .98 | .99 | 1 | .05 |
| Quantile+ComBat | .95 | .97 | .99 | .99 | .99 | .99 | .99 | .16 |
| EigenMS | .68 | .69 | .72 | .73 | .75 | .78 | .89 | .44 |
| VSN | .85 | .89 | .94 | .96 | .98 | .99 | 1 | .13 |
| Batch Normalizer | .94 | .96 | 1 | 1 | 1 | 1 | 1 | .91 |
| mixnorm | .98 | 1 | 1 | 1 | 1 | 1 | 1 | .06 |
| Downstream mixture modeling |  |  |  |  |  |  |  |  |
| Not normalized | .88 | .91 | .97 | 1 | 1 | 1 | 1 | .09 |
| Mean centering | .99 | 1 | 1 | 1 | 1 | 1 | 1 | .06 |
| Median scaling | .99 | 1 | 1 | 1 | 1 | 1 | 1 | .06 |
| Quantile | .87 | .91 | .97 | .99 | 1 | 1 | 1 | .06 |
| Quantile+ComBat | .95 | .97 | .99 | .99 | .99 | .99 | .99 | .17 |
| EigenMS | .69 | .70 | .73 | .75 | .77 | .80 | .89 | .48 |
| VSN | .87 | .91 | .96 | .98 | .99 | 1 | 1 | .13 |
| Batch Normalizer | .94 | .96 | 1 | 1 | 1 | 1 | 1 | .92 |
| mixnorm | .98 | 1 | 1 | 1 | 1 | 1 | 1 | .07 |
