# Supplementary figures and images for: Mixture model normalization for non-targeted gas chromatography/mass spectrometry metabolomics data
Source: BMC Bioinformatics. 2017 Feb 2;18:84. doi: 10.1186/s12859-017-1501-7 (PMC5290663; doi:10.1186/s12859-017-1501-7)

Figure S10

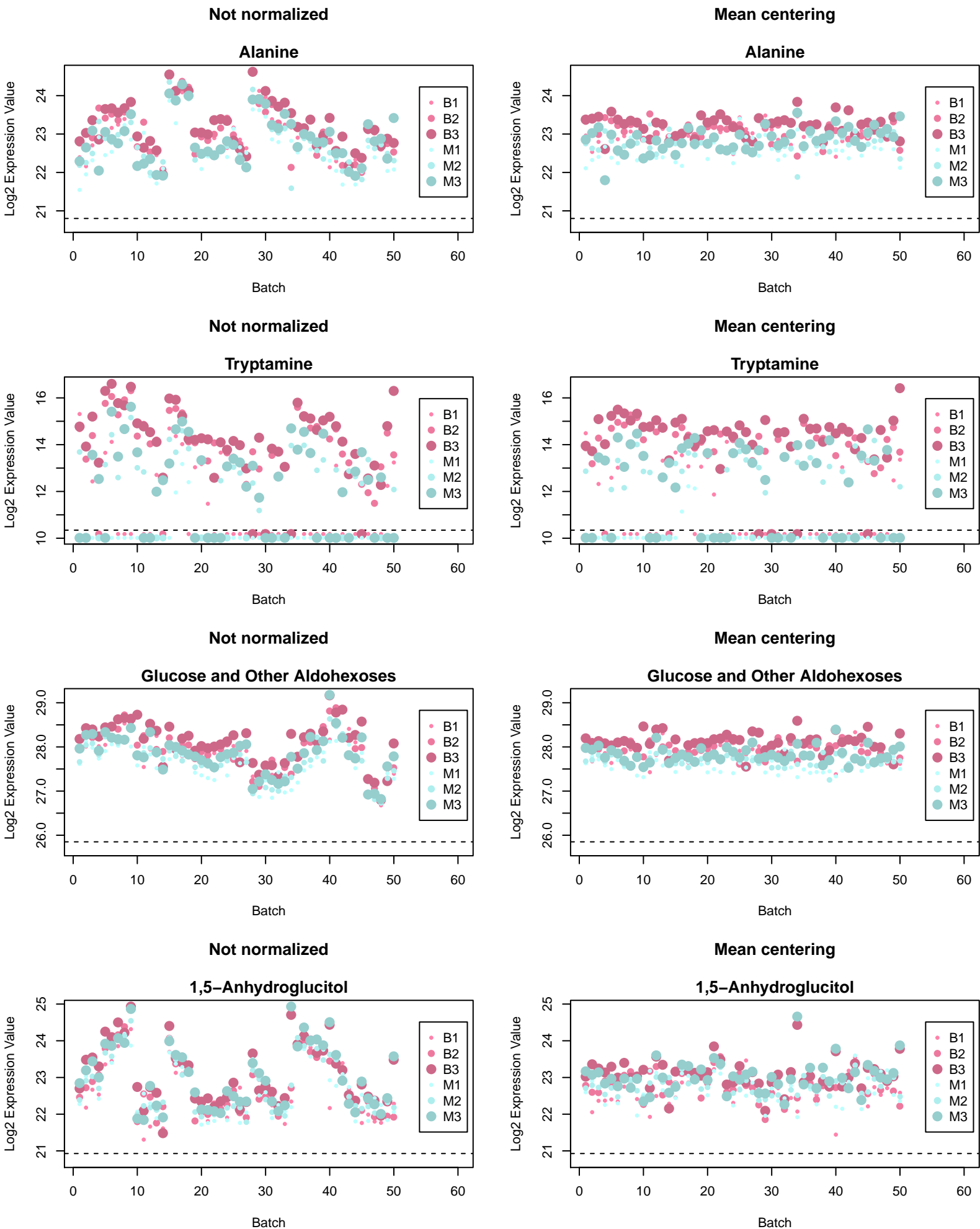

Figure S11

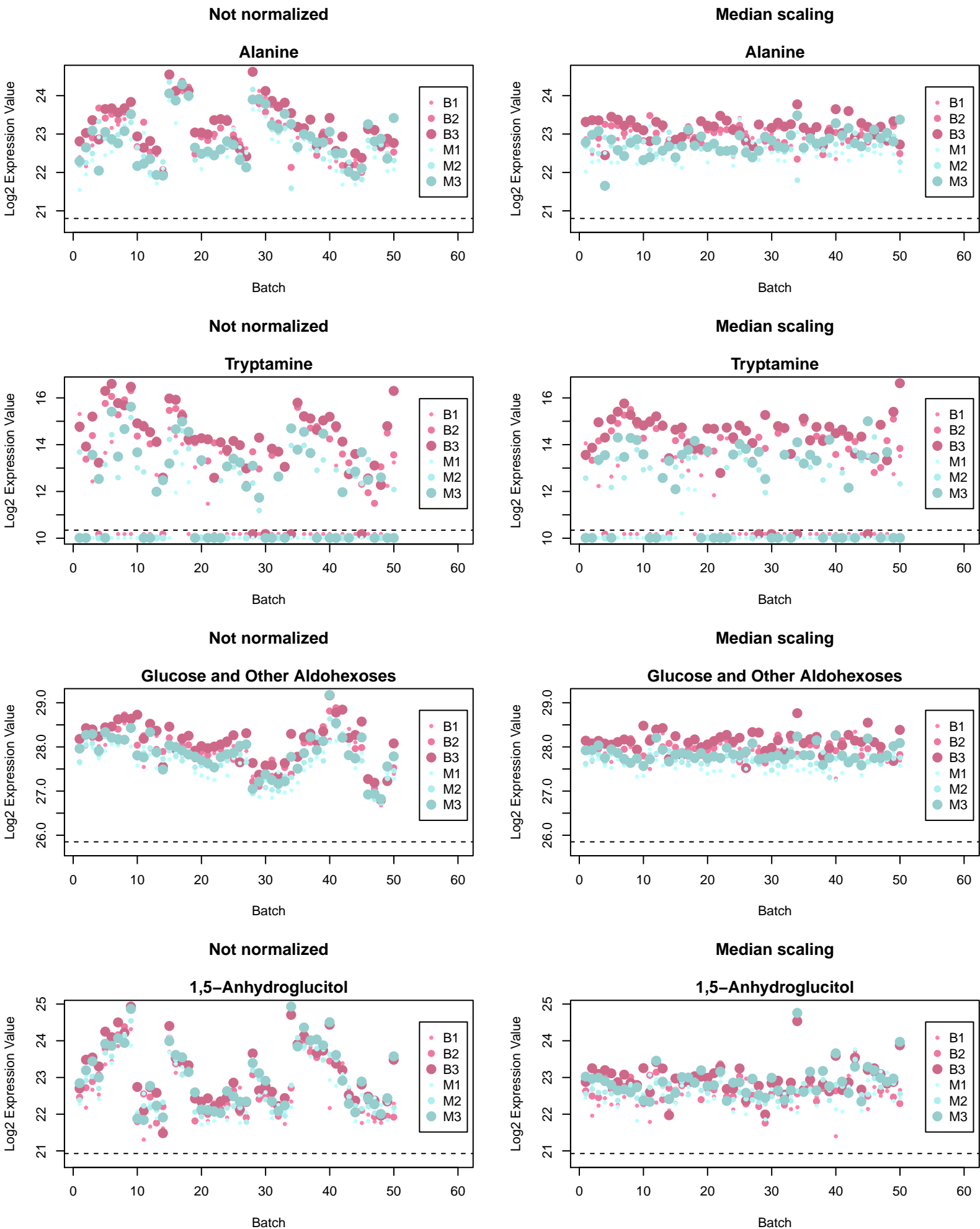

Figure S12

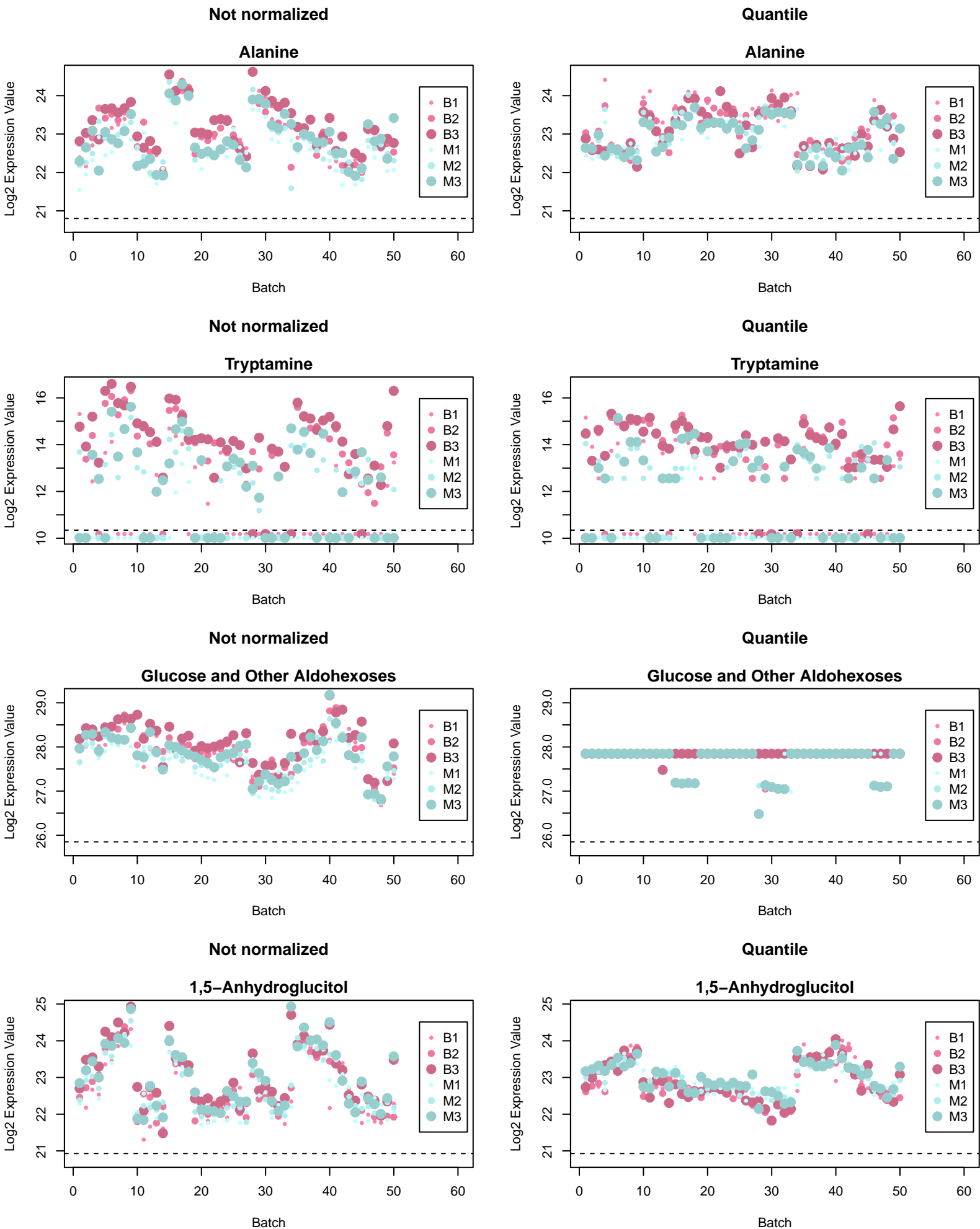

Figure S13

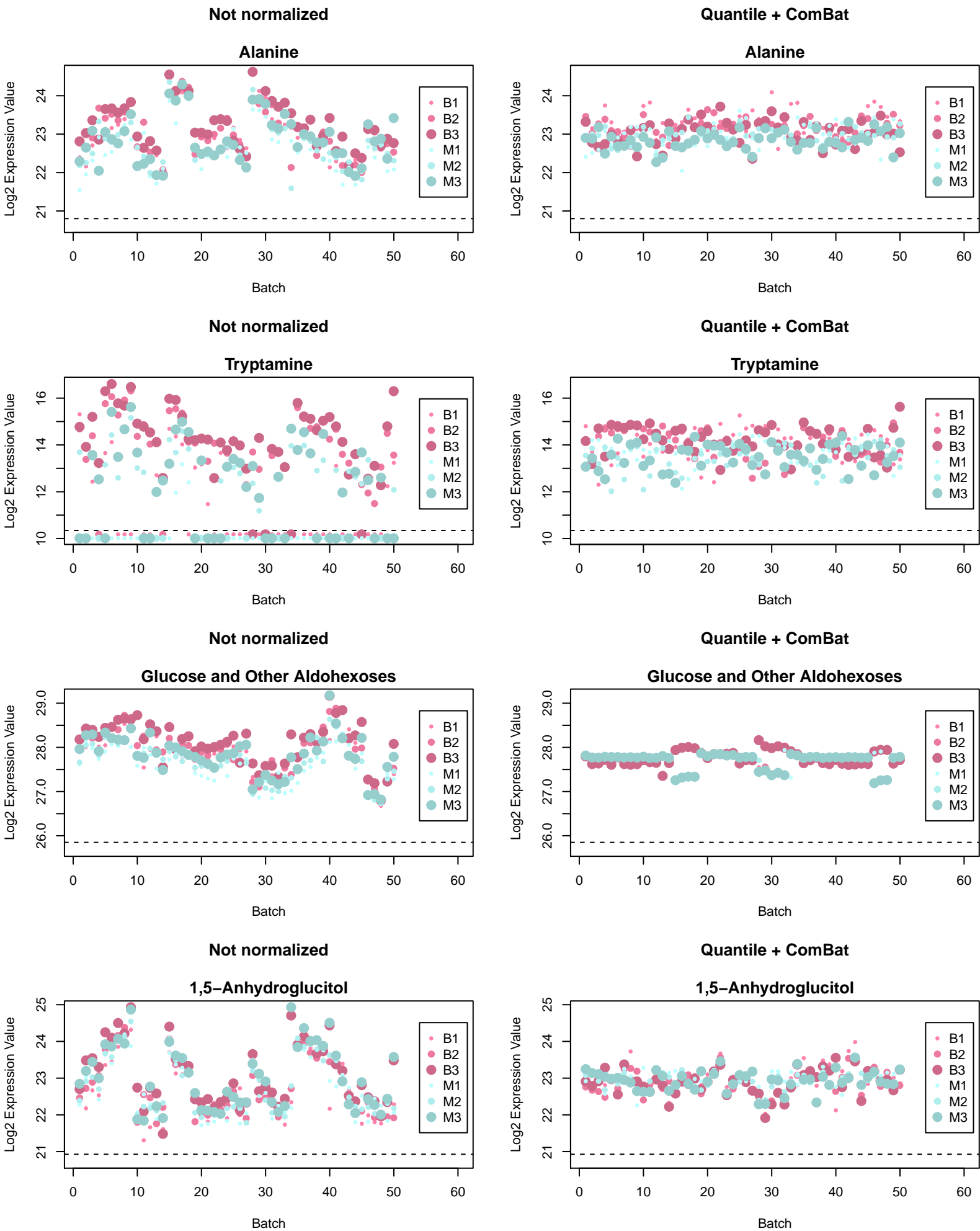

Figure S14

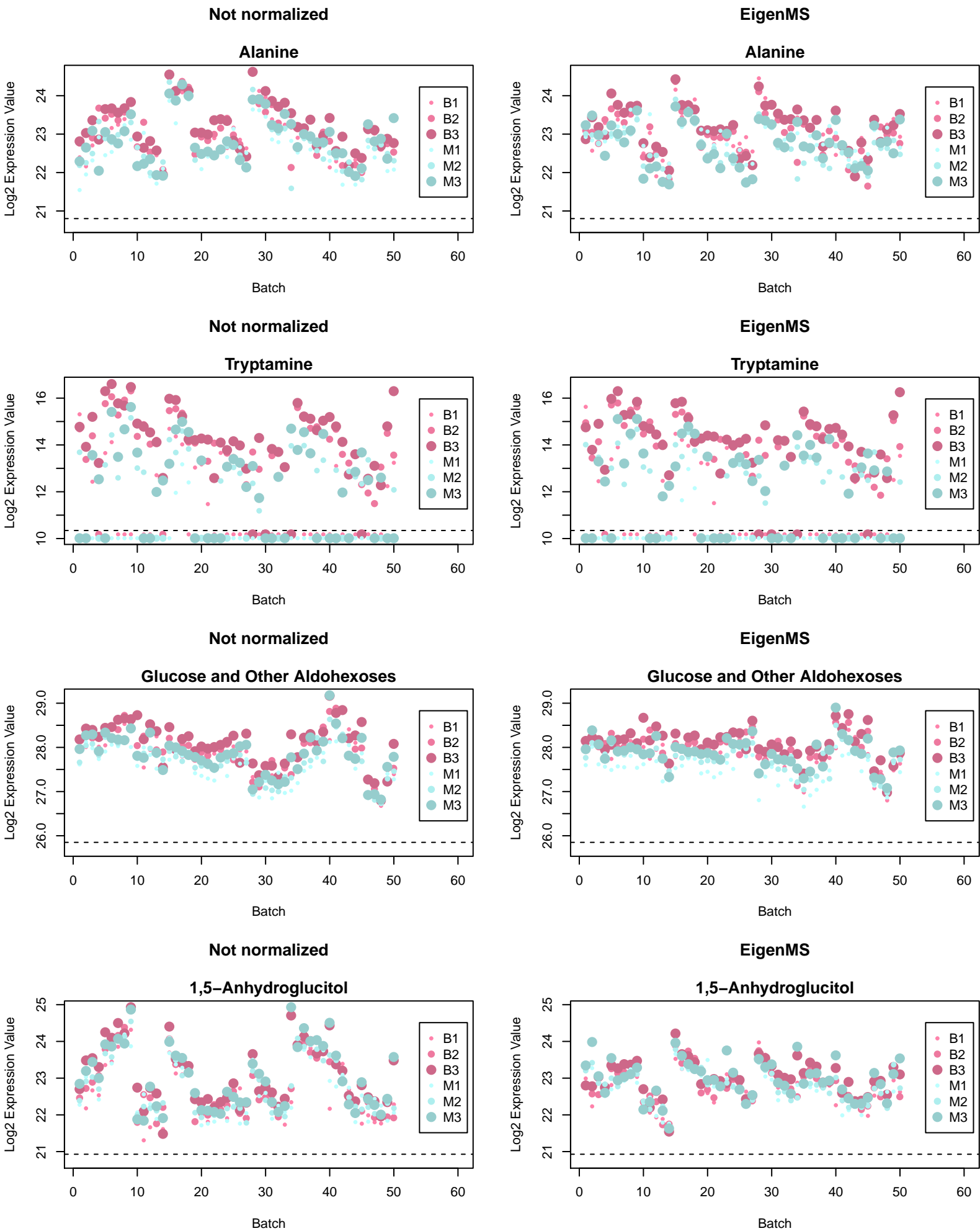

Figure S15

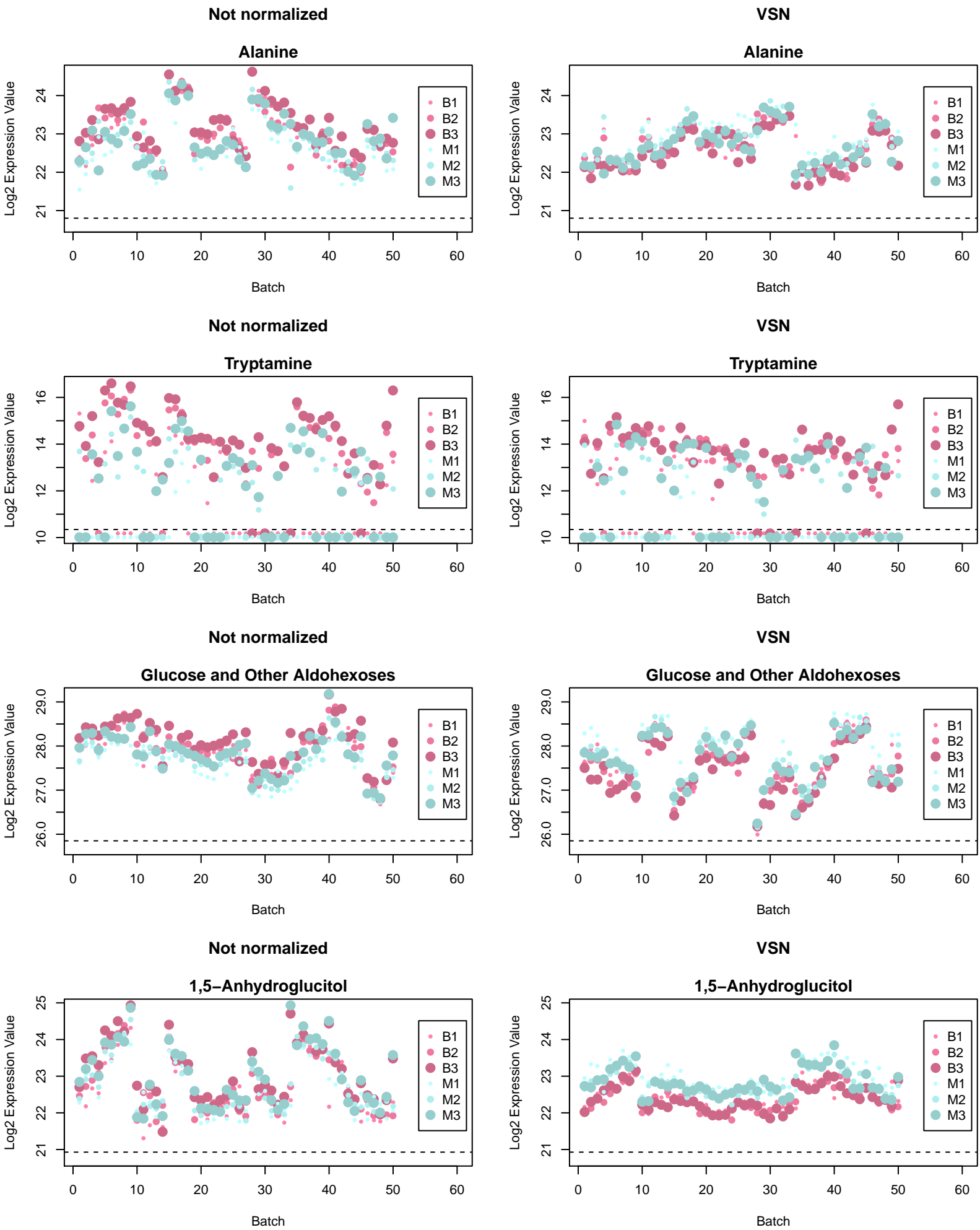

Figure S16

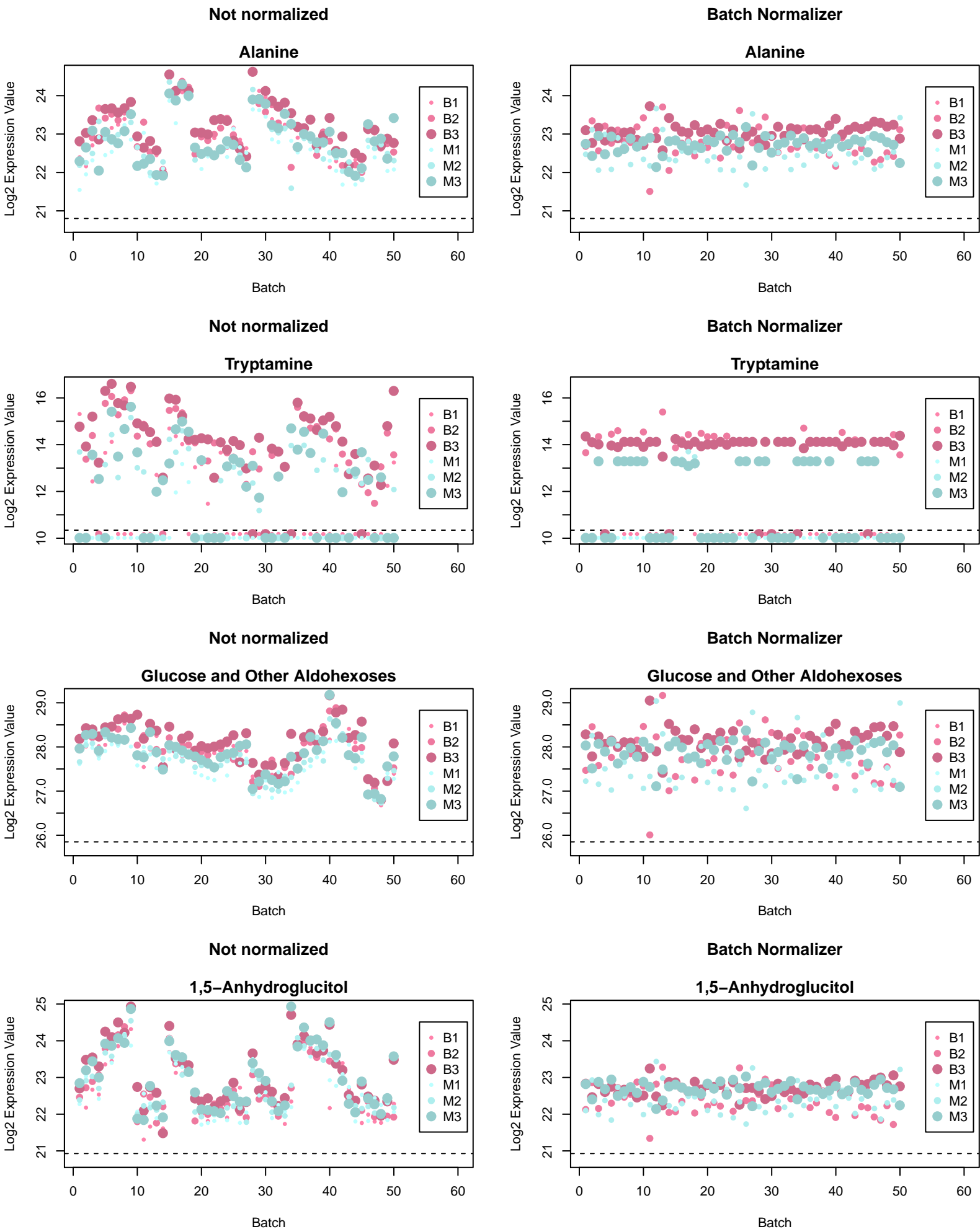

Supplement: Additional file 6: Figures S10–S16. — Plots of maternal and newborn QC HAPO Metabolomics samples prior to and following normalization for four selected metabolites. Figure S10. Plots of QC samples prior to and following mean centering. Figure S11. Plots of QC samples prior to and following median scaling. Figure S12. Plots of QC samples prior to and following quantile normalization. Figure S13. Plots of QC samples prior to and following quantile + ComBat. Figure S14. Plots of QC samples prior to and following EigenMS. Figure S15. Plots of QC samples prior to and following VSN. Figure S16. Plots of QC samples prior to and following Batch Normalizer. (PDF 774 kb) [file 12859_2017_1501_MOESM6_ESM.pdf]
