# Supplementary material for: Mixture model normalization for non-targeted gas chromatography/mass spectrometry metabolomics data
Source: BMC Bioinformatics. 2017 Feb 2;18:84. doi: 10.1186/s12859-017-1501-7 (PMC5290663; doi:10.1186/s12859-017-1501-7)
Supplement: Additional file 7: — Summary statistics for RSD for HAPO Metabolomics samples according to sample type prior to and following normalization. Lower RSD indicates better performance of the normalization method. RSD values for QC samples are expected to be lower than RSD for analytical samples since QCs are from common pools. (DOCX 75 kb) [file 12859_2017_1501_MOESM7_ESM.docx]

| **Additional File 5:** Summary statistics for RSD according to sample type prior to and following normalization. Lower RSD indicates better performance of the normalization method. RSD values for QC samples are expected to be lower than RSD for analytical samples since QCs are from common pools. | | | | | |
| --- | --- | --- | --- | --- | --- |
|  | **RSD % of individual metabolites across samples: mean (min, max)** | | | | |
|  | Maternal QC | Newborn QC | Maternal Fasting | Maternal 1-hour | Newborn Cord Serum |
| Not normalized | 5.8 (1.6, 14.6) | 5.8 (1.7, 12.0) | 6.0 (1.6, 13.5) | 6.0 (1.8, 14.1) | 6.4 (1.7, 13.2) |
| Mean centering | 3.7 (.7, 10.6) | 3.8 (.8, 9.3) | 4.1 (.9, 10.2) | 4.0 (1.0, 10.0) | 4.7 (.8, 13.0) |
| Median scaling | 3.7 (.7, 11.1) | 4.0 (.8, 9.7) | 4.2 (.9, 10.4) | 4.1 (1.0, 10.6) | 4.9 (.8, 13.4) |
| Quantile | 4.7 (1.0, 13.7) | 4.6 (.5, 12.3) | 5.0 (1.3, 12.3) | 5.0 (.9, 13.2) | 5.4 (.6, 12.9) |
| Quantile+ComBat | 3.3 (.7, 9.2) | 3.4 (.5, 10.1) | 3.6 (1.1, 8.5) | 3.7 (.6, 8.9) | 4.4 (.6, 12.4) |
| EigenMS | 4.8 (1.3, 11.4) | 4.9 (1.3, 10.8) | 5.2 (1.3, 11.5) | 5.2 (1.5, 11.2) | 5.6 (1.3, 13.2) |
| VSN | 4.5 (.8, 13.5) | 4.4 (1.0, 12.6) | 4.9 (1.3, 12.3) | 4.8 (1.4, 12.8) | 5.3 (1.6, 13.3) |
| Batch Normalizer | 1.7 (.6, 4.2) | 1.6 (.8, 4.5) | 5.8 (3.0, 15.6) | 5.7 (3.2, 14.0) | 6.1 (3.5, 14.9) |
| mixnorm | 2.9 (.5, 9.4) | 2.9 (.6, 7.3) | 4.9 (1.0, 15.4) | 4.8 (1.0, 13.4) | 5.3 (.8, 15.4) |
