# Supplementary material for: Mixture model normalization for non-targeted gas chromatography/mass spectrometry metabolomics data
Source: BMC Bioinformatics. 2017 Feb 2;18:84. doi: 10.1186/s12859-017-1501-7 (PMC5290663; doi:10.1186/s12859-017-1501-7)
Supplement: Additional file 8: — Summary statistics for pairwise Spearman correlation among HAPO Metabolomics QC samples prior to and following normalization. Higher pairwise Spearman correlation indicates better performance of the normalization method. (DOCX 51 kb) [file 12859_2017_1501_MOESM8_ESM.docx]

| **Additional File 6:** Summary statistics for pairwise Spearman correlation among QC samples prior to and following normalization. Higher pairwise Spearman correlation indicates better performance of the normalization method. | | |
| --- | --- | --- |
|  | **Pairwise Spearman correlation:**  **mean (min, max)** | |
|  | Maternal QC | Newborn QC |
| Not normalized | .93 (.77, 1.00) | .93 (.83, 1.00) |
| Mean centering | .96 (.87, 1.00) | .95 (.87, 1.00) |
| Median scaling | .96 (.87, 1.00) | .95 (.87, 1.00) |
| Quantile | .93 (.77, 1.00) | .93 (.83, 1.00) |
| Quantile+ComBat | .95 (.88, .99) | .94 (.89, .99) |
| EigenMS | .95 (.83, 1.00) | .95 (.85, 1.00) |
| VSN | .93 (.77, 1.00) | .93 (.83, 1.00) |
| Batch Normalizer | .99 (.95, 1.00) | .99 (.95, 1.00) |
| mixnorm | .98 (.93, 1.00) | .97 (.92, 1.00) |
