# Supplementary material for: Mixture model normalization for non-targeted gas chromatography/mass spectrometry metabolomics data
Source: BMC Bioinformatics. 2017 Feb 2;18:84. doi: 10.1186/s12859-017-1501-7 (PMC5290663; doi:10.1186/s12859-017-1501-7)
Supplement: Additional file 9: — Spearman correlation coefficients for non-targeted metabolites with conventional metabolites and targeted amino acid data in HAPO Metabolomics. (DOCX 23 kb) [file 12859_2017_1501_MOESM9_ESM.docx]

| **Additional File 7**: Spearman correlation coefficients for non-targeted metabolites with conventional metabolites and targeted amino acid data | | | | | | | | | |
| --- | --- | --- | --- | --- | --- | --- | --- | --- | --- |
|  | **Spearman correlation coefficient estimates:**  **maternal fasting, maternal 1-hour, newborn cord serum samples** | | | | | | | | |
|  | Not normalized | Mean centering | Median scaling | Quantile | Quantile + ComBat | EigenMS | VSN | Batch  Normalizer | mixnorm |
|  | **Conventional Metabolites** | | | | | | | | |
| β-Hydroxybutyrate | .75, .62, .87 | .89, .79, .94 | .87, .74, .93 | .79, .61, .93 | .87, .73, .95 | .75, .61, .87 | .85, .76, .93 | .70, .53, .78 | .91, .82, .95 |
| Lactate | .50, .47, .75 | .75, .80, .91 | .75, .79, .91 | .36, .44, .58 | .40, .47, .60 | .51, .52, .78 | .48, .45, .64 | .26, .23, .23 | .79, .81, .92 |
| Glycerol | .32, .21, .28 | .39, .26, .35 | .39, .27, .34 | .30, .23, .20 | .36, .22, .24 | .33, .21, .28 | .26, .16, .16 | .19, .10, .00 | .39, .29, .34 |
|  | **Amino Acids** | | | | | | | | |
| Alanine | .33, .36, .50 | .49, .58, .71 | .52, .60, .71 | .30, .27, .43 | .53, .47, .61 | .32, .40, .53 | .26, .25, .38 | .18, .14, .07 | .57, .64, .73 |
| Arginine | .12, .18, .34 | .27, .33, .52 | .26, .33, .50 | .13, .19, .45 | .16, .18, .55 | .11, .15, .38 | .08, .17, .51 | .21, .27, .56 | .30, .38, .52 |
| Asparagine | .39, .41, .54 | .44, .47, .63 | .45, .48, .64 | .48, .53, .59 | .49, .54, .34 | .36, .38, .59 | .52, .51, .62 | .34, .37, .57 | .45, .49, .64 |
| Glutamate | .49, .49, .71 | .67, .75, .86 | .67, .74, .86 | .63, .64, .81 | .76, .80, .88 | .53, .54, .77 | .69, .67, .84 | .57, .59, .72 | .69, .74, .87 |
| Glycine | .23, .26, .26 | .25, .32, .39 | .25, .32, .38 | .29, .36, .40 | .29, .36, .36 | .19, .26, .25 | .28, .34, .41 | .29, .22, .09 | .42, .46, .38 |
| Isoleucine | .28, .33, .31 | .46, .50, .53 | .45, .49, .52 | .32, .36, .30 | .36, .37, .33 | .19, .27, .31 | .31, .33, .35 | .11, .12, .10 | .52, .56, .55 |
| Leucine | .31, .29, .35 | .49, .49, .41 | .49, .50, .39 | .38, .31, .32 | .46, .39, .28 | .29, .32, .38 | .33, .25, .33 | .18, .15, .10 | .49, .53, .39 |
| Methionine | .16, .19, .19 | .24, .27, .37 | .27, .28, .38 | .19, .28, .21 | .19, .31, .33 | .18, .17, .18 | .27, .21, .25 | .12, .10, .04 | .25, .28, .31 |
| Ornithine | .30, .34, .41 | .50, .55, .66 | .47, .52, .63 | .34, .36, .53 | .53, .49, .67 | .42, .49, .42 | .45, .44, .61 | .30, .26, .28 | .53, .54, .64 |
| Phenylalanine | .24, .27, .30 | .46, .56, .50 | .47, .57, .51 | .31, .35, .31 | .47, .56, .45 | .14, .27, .29 | .36, .37, .38 | .18, .21, .10 | .51, .55, .51 |
| Proline | .40, .43, .35 | .59, .62, .58 | .60, .64, .59 | .42, .45, .29 | .57, .60, .45 | .38, .44, .39 | .40, .40, .26 | .34, .31, .06 | .64, .66, .57 |
| Serine | .32, .32, .25 | .46, .48, .60 | .46, .49, .60 | .40, .42, .50 | .49, .49, .60 | .25, .28, .27 | .47, .47, .55 | .22, .18, .17 | .46, .48, .55 |
| Tyrosine | .09, .09, .28 | .35, .37, .51 | .35, .37, .52 | .16, .15, .38 | .35, .35, .49 | .07, .08, .17 | .18, .17, .41 | .19, .16, .16 | .33, .34, .49 |
| Valine | .33, .35, .31 | .56, .60, .58 | .57, .61, .58 | .34, .34, .36 | .52, .50, .51 | .25, .29, .24 | .25, .31, .31 | .16, .10, .13 | .60, .63, .60 |
|  | **Spearman correlation estimate summaries for all sample types: mean (min, max)** | | | | | | | | |
| Maternal fasting | .33 (.09, .75) | .49 (.24, .89) | .49 (.25, .87) | .36 (.13, .79) | .46 (.16, .87) | .30 (-.07, .75) | .38 (.08, .85) | .27 (.11, .70) | .52 (.25, .91) |
| Maternal 1-hour | .33 (.09, .62) | .51 (.26, .80) | .51 (.27, .79) | .37 (.15, .64) | .46 (.18, .80) | .33 (.08, .61) | .37 (.16, .76) | .24 (.10, .59) | .54 (.28, .82) |
| Newborn cord serum | .41 (.19, .87) | .59 (.35, .94) | .59 (.34, .93) | .45 (.20, .93) | .51 (.24, .95) | .42 (.17, .87) | .47 (.16, .93) | .25 (.00, .78) | .59 (.31, .95) |
