# Supplementary material for: Mixture model normalization for non-targeted gas chromatography/mass spectrometry metabolomics data
Source: BMC Bioinformatics. 2017 Feb 2;18:84. doi: 10.1186/s12859-017-1501-7 (PMC5290663; doi:10.1186/s12859-017-1501-7)
Supplement: Additional file 10: — MetaboAnalyst 3.0 pathway results for each normalization method and both linear regression and mixture model analysis of phenotypic associations in HAPO Metabolomics. (DOCX 140 kb) [file 12859_2017_1501_MOESM10_ESM.docx]

| **Additional File 8:** MetaboAnalyst 3.0 pathway results for each normalization method and both linear regression and downstream mixture model analysis of phenotypic associations | | | | |
| --- | --- | --- | --- | --- |
|  | Linear Regression Analysis | | Downstream Mixture Model Analysis | |
|  | Pathway name | p | Pathway name | p |
| Not | Pentose phosphate pathway | 0.002 | Pentose phosphate pathway | 0.002 |
| normalized | Galactose metabolism | 0.003 | Galactose metabolism | 0.003 |
|  | Fatty acid biosynthesis | 0.004 | Fatty acid biosynthesis | 0.004 |
| Mean Center | Alanine, aspartate and glutamate metabolism | 0.0001 | Glycolysis or Gluconeogenesis | 0.0001 |
|  | Glycolysis or Gluconeogenesis | 0.0003 | Pentose phosphate pathway | 0.0002 |
|  | Pentose phosphate pathway | 0.0003 | Taurine and hypotaurine metabolism | 0.002 |
|  | Citrate cycle (TCA cycle) | 0.003 | Alanine, aspartate and glutamate metabolism | 0.003 |
|  | Pyruvate metabolism | 0.009 | Pyruvate metabolism | 0.006 |
|  | Butanoate metabolism | 0.013 | Galactose metabolism | 0.009 |
|  | Galactose metabolism | 0.014 | Fatty acid biosynthesis | 0.013 |
|  | Ascorbate and aldarate metabolism | 0.017 | Starch and sucrose metabolism | 0.014 |
|  | Fatty acid biosynthesis | 0.020 | Cysteine and methionine metabolism | 0.017 |
|  | Glyoxylate and dicarboxylate metabolism | 0.021 |  |  |
|  | Cysteine and methionine metabolism | 0.026 |  |  |
| Median scaling | Alanine, aspartate and glutamate metabolism | 0.0001 | Alanine, aspartate and glutamate metabolism | 0.0001 |
|  | Glycolysis or Gluconeogenesis | 0.0002 | Glycolysis or Gluconeogenesis | 0.0002 |
|  | Pentose phosphate pathway | 0.0002 | Pentose phosphate pathway | 0.0002 |
|  | Citrate cycle (TCA cycle) | 0.003 | Citrate cycle (TCA cycle) | 0.003 |
|  | Pyruvate metabolism | 0.007 | Pyruvate metabolism | 0.007 |
|  | Butanoate metabolism | 0.011 | Butanoate metabolism | 0.011 |
|  | Galactose metabolism | 0.012 | Galactose metabolism | 0.012 |
|  | Ascorbate and aldarate metabolism | 0.014 | Ascorbate and aldarate metabolism | 0.014 |
|  | Fatty acid biosynthesis | 0.016 | Fatty acid biosynthesis | 0.016 |
|  | Glyoxylate and dicarboxylate metabolism | 0.017 | Glyoxylate and dicarboxylate metabolism | 0.017 |
|  | Cysteine and methionine metabolism | 0.021 | Cysteine and methionine metabolism | 0.021 |
| Quantile | Pentose phosphate pathway | 0.0001 | Pentose phosphate pathway | 0.0001 |
|  | Galactose metabolism | 0.006 | Galactose metabolism | 0.006 |
|  | Fatty acid biosynthesis | 0.008 | Fatty acid biosynthesis | 0.008 |
| Quantile + | Pentose phosphate pathway | 0.010 | Pentose phosphate pathway | 0.010 |
| Combat | Fatty acid biosynthesis | 0.024 | Fatty acid biosynthesis | 0.024 |
|  | Starch and sucrose metabolism | 0.024 | Starch and sucrose metabolism | 0.024 |
| EigenMS | Pentose phosphate pathway | 0.002 | Pentose phosphate pathway | 0.002 |
|  | Galactose metabolism | 0.003 | Galactose metabolism | 0.003 |
|  | Fatty acid biosynthesis | 0.004 | Fatty acid biosynthesis | 0.004 |
| VSN | Pentose phosphate pathway | 0.0001 | Pentose phosphate pathway | 0.0001 |
|  | Galactose metabolism | 0.004 | Galactose metabolism | 0.004 |
|  | Fatty acid biosynthesis | 0.006 | Fatty acid biosynthesis | 0.006 |
| Batch Normalizer | Pentose phosphate pathway | 0.002 | Glycolysis or Gluconeogenesis | 0.004 |
|  | Fatty acid biosynthesis | 0.006 | Pentose phosphate pathway | 0.005 |
|  | Taurine and hypotaurine metabolism | 0.049 | Pyruvate metabolism | 0.005 |
|  | Citrate cycle (TCA cycle) | 0.049 | Fatty acid biosynthesis | 0.011 |
| mixnorm | Glycolysis or Gluconeogenesis | 0.0002 | Glycolysis or Gluconeogenesis | 0.0002 |
|  | Pentose phosphate pathway | 0.0002 | Pentose phosphate pathway | 0.0002 |
|  | Taurine and hypotaurine metabolism | 0.002 | Taurine and hypotaurine metabolism | 0.003 |
|  | Alanine, aspartate and glutamate metabolism | 0.003 | Alanine, aspartate and glutamate metabolism | 0.004 |
|  | Pyruvate metabolism | 0.006 | Pyruvate metabolism | 0.007 |
|  | Galactose metabolism | 0.009 | Galactose metabolism | 0.012 |
|  | Fatty acid biosynthesis | 0.013 | Fatty acid biosynthesis | 0.016 |
|  | Starch and sucrose metabolism | 0.014 | Starch and sucrose metabolism | 0.017 |
|  | Cysteine and methionine metabolism | 0.017 | Cysteine and methionine metabolism | 0.021 |
